# The Impacts of Green Innovation Input and Channel Service in a Dual-Channel Value Chain

**DOI:** 10.3390/ijerph16224566

**Published:** 2019-11-18

**Authors:** Qiuxiang Li, Xingli Chen, Yimin Huang, Huangbao Gui, Shengyang Liu

**Affiliations:** 1 Institute of Management Science and Engineering, Henan University, Kaifeng 475004, China; lqxkycg@henu.edu.cn; 2 School of Business, Henan University, Kaifeng 475004, China; 104754181123@vip.henu.edu.cn; 3 School of Management & Economics, North China University of Water Resources and Electric Power, Zhengzhou 450046, China

**Keywords:** price game, green innovation input, channel service, value chain, stability

## Abstract

This paper constructs a dual-channel value chain composed of one altruistic manufacturer and one altruistic retailer, where the manufacturer makes green innovation input for green products and sells its green products to its customers through both the direct channel and the traditional channel, the retailer provides channel service for customers and sells green products through the traditional channel. We consider two scenarios in which the manufacturer and the retailer make decentralized and centralized decisions, respectively. We develop two dynamic game models for the two scenarios and analyze the dynamic behaviors of the two dynamic game models using bifurcation diagram, LLE (largest Lyapunov exponent) and attraction of basin, etc. We find that the stability region in decentralized decision model is greater than that of centralized decision, and narrow with increase of service value, green innovation input. In the decentralized decision model, the stability of the system decreases with the altruistic behavior increasing. With the price adjustment speed increasing, the dual-channel green value chain system enters into chaotic state through flip bifurcation or N-S bifurcation. In the stable state, the manufacturer and the retailer can obtain the maximum utility with the appropriate value of green innovation input. In the chaotic state, the utilities of the manufacturer and retailer are greatly affected and significantly reduced. This study will provide good guidance for sustainable development decision-making of dual-channel green value chain.

## 1. Introduction

In 2017, the proposal of high-quality development indicates that China’s economy has changed from a high-speed growth stage to a high-quality development stage. In this context, consumers have higher requirements for the quality of commodities. They not only care about the price and quality of commodities, but also have a growing preference for green products. This concept of consumers enables enterprises to produce a variety of green products and prompt manufacturers to make green technological innovation inputs in green products. From the perspective of environmental protection, green products gradually enter the market and become one of the ways for enterprises to obtain profits.

In a dual-channel value chain, many enterprises are currently investing in green innovation to make products more green and intelligent, such as Huawei and Samsung. In addition, many retailers invest in retail services to meet consumer demand for high-quality retail services, such as Haier and Jingdong. The behaviors of the manufacturer and the retailer will affect the stability of the value chain system. The impact of green innovation input and channel service on the stability of dual-channel supply system is an interesting topic. How does it affect the stability of dual-channel value chain system when the manufacturer invests in green innovation and the retailer invests in channel service? How does service value and green innovation input affect price decision and system profit? Whether the enterprise can provide more green innovation input and channel services to obtain more profits?

Based on these problems, this paper establishes two price game models considering the factors of green innovation input and channel service in two scenarios separately (decentralized decision scenario and centralized decision scenario). Using game theory and nonlinear dynamics theory, we discuss the equilibrium points and the complex dynamic behaviors of two price game models; and we study the effects of green innovation and channel service on optimal pricing, stability and utility of dual-channel value chain system. This paper can provide some management theory suggestions and references for enterprises which can help enterprises make better decisions.

This paper is organized as follows: Section 2 is a literature review. Section 3 presents the problem description and model assumptions. In Section 4, the decentralized game model is established, and the static and the dynamic analyses of the model are given respectively. Section 5 establishes centralized decision model and studies the complexity of the model by numerical simulation. The conclusions are drawn in Section 6.

## 2. Literature Review

Many scholars have studied the influence of technological innovation on enterprises in the literature [1,2,3]. González and Pazó [4] proposed that a firm performs R&D (Research and Development) activities only when optimal level of R&D expenditure is higher than a threshold. By means of a differential game approach, Hasnas et al. [5] analyzed the case of substitutability in a Cournot duopoly where knowledge spillover was endogenously determined via the R&D process. Du et al. [6] thought that fairness concern could promote and coordinate the value chain members to invest more in sustainable development of green technology innovation. Some scholars have studied the green technological innovation of enterprises using empirical research methods [7]. Yuan and Xiang [8] employed the panel data of Chinese manufacturing industries during 2003–2014 and examined the effects of environmental regulation on industrial innovation and green development. Qu et al. [9] investigated the role of regional formal institutions in the innovation process of Chinese enterprises. Mensah et al. [10] found environmental related budget and taxes are worthwhile in the pursuit of green growth from the dominant negative coefficient values, which was useful for policy-makers and industries in the pursuit of green growth globally. Lin and Zhu [11] explored the response of renewable energy technological innovation to intensive CO_2_ emissions and analyzed the effect of energy price and R&D investment on this green innovation process. Lee et al. [12] identified the relationship between green value chain management practices and technological innovation in manufacturing firms, found that green purchasing and cooperation with customers did not have a significant positive correlation with technological innovation. Lee et al. [13] found that through proper implementation of SCM practices, firms are able to achieve better green technological innovation performance. According to the above literature, scholars mainly studied the influence of green innovation input on enterprise development, the influencing factors of enterprise innovation activities and so on.

The above literature demonstrated the impact of green innovation activities on enterprise development. However, different subsidy methods may affect enterprises’ enthusiasm for green innovation and change enterprises’ profits, so scholars have also studied the issue of innovation subsidies. Guo et al. [14] investigated the effects of government R&D projects on firm’s innovation outputs. Similarly, Ma et al. [15] found that the degree of government innovation subsidy showed an effect on the stability and entropy of the dynamic duopoly game model in two cases. Sabri et al. [16] developed a framework capturing the dynamics changes between innovation implementation and allocation decisions. Considering process innovation that reduced marginal costs, Lambertini and Mantovani [17] investigated the timing of product adoption and process innovation using a differential game where firms may invest in both activities. On enterprise innovation cooperation, different cooperation strategies will lead to different profit distribution among participants. Kim [18] researched a particular value chain situation in which the manufacturer coordinated and supported supplier’s innovation. Using system dynamics method, the influence of innovation activities on the profit of dynamic system is studied [19]. Santos et al. [20] provided a precise analysis about the selection process of applications submitted for public support, and found that firms with approved applications were those that expect to invest more and forecast a higher increase. Bai et al. [21] explored the impacts of government R&D subsidies on the green innovation of energy-intensive firms, and showed that government R&D subsidies increase the green innovation of energy-intensive firms. Liu et al. [22] found the level of regional economic development moderates the impact of government subsidies and there is more significant for non-state-owned enterprises than state-owned ones. The results showed that the coordination could improve the profitability of manufacturing firms. It might not be attractive to the supplier unless the supply cost reduction could ultimately increase the market demand to a certain extent.

The above literature has studied the impact of innovation input on cooperation among enterprises, government subsidy policy, and price decision-making of the value chain. However, few studies have looked at the influence of the manufacturer’s green innovation input on the stability and the profit of a dual-channel value chain. In the dual-channel value chain management, when the value chain is in a stable state, participants can make compete in an orderly market and obtain stable profits; when the value chain is in an unstable state, participants’ decisions will become disorderly and unpredictable, and the firm’s profits will be greatly affected. Aydin and Parker [23] developed a game model in a two-tier value chain and studied the diffusion effect of innovation and technology in competitive value chains. Song et al. [24] considered that both innovation and advertising contribute to the product demand, and investigated the optimal equilibriums of channel members under two different game structures, however the interaction between innovation and service was not considered. Kirikkaleli and Ozun [25] explored the linkages between innovation capacity, business sophistication, and macroeconomic stability within OECD (Organization for Economic Cooperation and Development) countries, and found innovation capacity positively leads to advanced business sophistication. Carlos et al. [26] compared the innovation output indicators of Spanish knowledge-intensive service firms with those of other categories of non-knowledge-intensive service firms and manufacturing firms, found the main determinants of innovation output are the following: cooperation with other partners to innovate, R&D intensity, and the size of the firm. Wei and Chan [27] found the cooperative stability of China’s satellite industry military-civilian collaborative innovation was positively related to the cooperation revenue, liquidated damages, and government incentives, and negatively related to basic income, R&D costs, information communication costs, etc. Kumar et al. [28] proposed a nonlinear mathematical model for innovation diffusion with stage structure, and found that Hopf bifurcation would occur when the evaluation period (time delay) passed through a critical value.

There are some studies of participant altruistic preference. Chen and Chen [29] studied the behavioral decision of green innovation efforts which consider the influence of participants’ altruistic preference under two decision structures. The results showed that altruistic preferences had a positive effect on participants’ effort behavior. Cheng et al. [30] established a value chain pricing and emission reduction decision-making model with altruistic preference of the manufacturer and retailer and analyzed the influence of altruistic preference attributes on the decision-making of the value chain members. Xu and Wang [31] proposed a competitive-cooperative game models of the dual-channel value chain based on altruistic behavior. Shi and Ma [32] found that the evolutionary direction of altruistic behavior is determined by the sensitiveness and the learning abilities of both members of value chains. Fan et al. [33] incorporated the retailer’s altruistic behavior into the low-carbon supply chain considering consumers’ low-carbon preference and found that the retailer’s altruistic behavior and consumers’ low-carbon preference narrow the stability region of the system. Huang et al. [34] addressed the pricing decisions and optimal greenness of decision makers, and analyzed the impact of altruistic preferences on decision-making and profits of supply chain. Liu et al. [35] found that supply chain coordination could not be achieved when both the logistics service integrator and functional logistics service provider had altruistic preferences, achieved coordination by designing the ex-post payment contract, revenue sharing, and franchise fee’ contract.

In general, service has become an important factor affecting consumer’s channel choice and profit in a value chain [36,37,38,39]. Ren et al. [40] found that allowing customers to return products would increase the costs of the manufacturer and network retailer and affect the sales price of product. Pei and Yan [41] supported retail service as an effective incentive to coordinate the dual-channel distribution. Li and Li [42] designed a dual-channel value chain considering channel competition and the value-added service provided by retailer. Protopappa-Sieke and Sieke [43] developed the optimal two-period inventory allocation policies under multiple service level contracts in view of the fact that optimal inventory allocation had a significant impact on profits in the retail industry. Zhou et al. [44] investigated how free riding affected the two members’ pricing/service strategies and profits when the dual channels used the differential and non-differential pricing scenarios, respectively. Sadjadi et al. [45] introduced the Stackelberg game where two manufacturers and one retailer competed simultaneously considering price, service and simple price discount contract. The preliminary results showed that the service and the price discount contract could improve the performance of value chain. Chen et al. [46] investigated the interaction between providing free after-sales service and contract types (wholesale price contracts and consignment contracts with revenue sharing) in a two-echelon value chain with one manufacturer and one retailer facing random demand. Jena and Sarmah [47] studied the coopetition of price and service between the two remanufacturing firms. Zhang and Wang [48] investigated two dynamic pricing strategies in a dual-channel value chain in which the retailer focused on the influence of service value on the price decisions. Ma et al. [49] examined the optimal decisions of a dual-channel game model considering the retailing service investment, and analyzed how adjustment speed of service inputs affected the system complexity and market performance. Finally, Ghosh [50] discussed the optimal pricing strategy of a two-echelon value chain consisting of one manufacturer and two retailers. The above literatures studied the impact of service level on the channel conflict and value chain profits in a dual-channel value chain.

## 3. Basic Model

**Figure 1:**
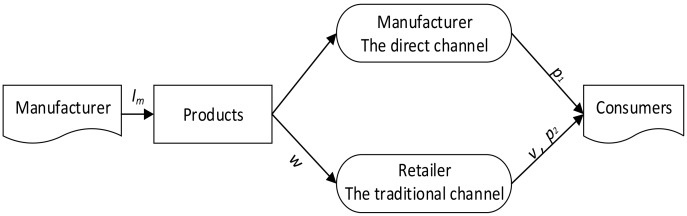
The dual-channel value chain system.

This paper explores a dual-channel value chain which includes a manufacture, a retailer, and consumers, as shown in Figure 1. The manufacturer carries out green innovation investment ($I_{m}$) in production, sells the green products through both the direct channel with the price of $p_{1}$ and the traditional channel with the price of w; the retailer carries out service input (v) in traditional channel and sells the green products to consumers with the price of $p_{2}$. The manufacturer and retailer show altruistic preferences in decision-making.

The model developed in this paper is based on the following assumptions:

(1) The manufacturer and the retailer face the same customer demand in a certain proportion θ and make price competition in the same market.
(2) Both the manufacturer and the retailer have bounded rational behavior and altruistic preference behavior.
(3) Green innovation input and service cost function are quadratic functions.

The notations used in this paper are shown in Table 1:

The green innovation investment in products can improve customer demand for products. Considering the marginal decreasing effect of green innovation input on customer demand, the following equation is obtained:

(1) $$Q=\frac{1}{\sqrt{\varepsilon }}\sqrt{I_{m}}.$$

In market competition, the retailer cares about channel service in product sales. According to literature [39], let us use a strictly convex service function to depict the relationship between service value and its related cost:

(2) $$c_{r}=\eta \frac{v^{2}}{2}.$$

Here, assuming $v+w<p_{2}$ to ensure the retailer is profitable.

Considering that customer demand is influenced by price, green innovation input, and service value, the demand functions of the manufacturer and the retailer are described as follows [51]:

(3) $$D_{m}=\theta a-b_{1}p_{1}+\beta_{1}\left(p_{2}-v\right)+\theta \frac{1}{\sqrt{\varepsilon }}\sqrt{I_{m}}$$

(4) $$D_{r}=\left(1-\theta \right)a-b_{2}\left(p_{2}-v\right)+\beta_{2}p_{1}+\left(1-\theta \right)\frac{1}{\sqrt{\varepsilon }}\sqrt{I_{m}}.$$

Thus, the total demand of the dual-channel value chain is expressed as follows:

(5) $$D^{T}=D_{m}+D_{r}=a-\left(b_{1}-\beta_{2}\right)p_{1}-\left(b_{2}-\beta_{1}\right)\left(p_{2}-v\right)+\frac{1}{\sqrt{\varepsilon }}\sqrt{I_{m}}.$$

Based on the actual market situation, parameters in this paper should satisfy the following conditions:

(6) $$\left\{\begin{array}{l} 0<c<w<p_{1}\\ w+c_{r}<p_{2}\\ v+w<p_{2}\\ D_{m}>0,D_{r}>0 \end{array}\right..$$

From inequality (6), we can obtain the following inequality (7):

(7) $$\left\{\begin{array}{c} 0<c<w<p_{1}<\frac{\theta ab_{2}+\left(1-\theta \right)a\beta_{1}+\left(1-\theta \right)\frac{\beta_{1}}{\sqrt{\varepsilon }}\sqrt{I_{m}}+\theta \frac{b_{2}}{\sqrt{\varepsilon }}\sqrt{I_{m}}}{b_{1}b_{2}\left(1-\beta_{1}\beta_{2}\right)}\\ 0<w+c_{r}<p_{2}<\frac{\left(1-\theta \right)ab_{1}+\theta a\beta_{2}+\theta \frac{\beta_{2}}{\sqrt{\varepsilon }}\sqrt{I_{m}}+\left(1-\theta \right)\frac{b_{1}}{\sqrt{\varepsilon }}\sqrt{I_{m}}}{b_{1}b_{2}\left(1-\beta_{1}\beta_{2}\right)}+v \end{array}\right..$$

The manufacturer’s profit function is as follows:

(8) $$\pi_{m}=\left(p_{1}-c\right)D_{m}+\left(w-c\right)D_{r}-I_{m}.$$

Namely, the profit of the retailer is as follows:

(9) $$\pi_{r}=\left(p_{2}-w-c_{r}\right)D_{r}.$$

In market competition, the decision makers care not only about their own profits, but also about the profits of other participants, which shows an altruistic preference behavior. Altruistic behavior is defined as self-conscious and voluntary behavior that benefits others and has no obvious selfish motivation. It includes the following characteristics: (1) for the purpose of helping others; (2) not expecting spiritual or material rewards, such as honor or prize; (3) voluntary; (4) altruistic behavior may suffer losses. For the stability and durability of the value chain, when making decisions, the manufacturer considers not only its own interests, but also the interests of the retailer, and vice versa. On the basis of existing results the utility functions of the manufacturer and retailer are as follows [52,53]:

(10) $$\left\{\begin{array}{l} U_{m}=\pi_{m}+\gamma \pi_{r}\\ U_{r}=\pi_{r}+\varphi \pi_{m} \end{array}\right..$$

Namely

(11) $$\left\{\begin{array}{l} \begin{array}{ll} U_{m}= & \left[\left(w-c\right)+\gamma \left(p_{2}-w-c_{r}\right)\right]\left[\left(1-\theta \right)a-b_{2}\left(p_{2}-v\right)+\beta_{2}p_{1}+\left(1-\theta \right)\frac{1}{\sqrt{\varepsilon }}\sqrt{I_{m}}\right]\\ & +\left(p_{1}-c\right)\left[\theta a-b_{1}p_{1}+\beta_{1}\left(p_{2}-v\right)+\theta \frac{1}{\sqrt{\varepsilon }}\sqrt{I_{m}}\right]-I_{m}\; \end{array}\\ \begin{array}{ll} U_{r}= & \left[\left(p_{2}-w-c_{r}\right)+\varphi \left(w-c\right)\right]\left[\left(1-\theta \right)a-b_{2}\left(p_{2}-v\right)+\beta_{2}p_{1}+\left(1-\theta \right)\frac{1}{\sqrt{\varepsilon }}\sqrt{I_{m}}\right]\\ & +\varphi \left(p_{1}-c\right)\left[\theta a-b_{1}p_{1}+\beta_{1}\left(p_{2}-v\right)+\theta \frac{1}{\sqrt{\varepsilon }}\sqrt{I_{m}}\right]-{\varphi I}_{m} \end{array} \end{array}\right.$$

where $\gamma ,\varphi \in \left(0,\;1\right)$. The manufacturer is completely self-interested when γ=0; the manufacturer is a decision maker of altruistic preference when 0 <γ< 1. Namely, the higher the value of γ is, the higher the degree of altruistic preference of the manufacturer is. The decision goals of the manufacturer and retailer are to maximize their utilities.

## 4. Decentralized Game Model

In this model, the manufacturer and retailer hold symmetric channel power and make decisions simultaneously to maximize their utility, respectively. Making the first derivative of $U_{m}$ and $U_{r}$ with respect to $p_{1}$ and $p_{2}$, the reaction functions of the manufacturer and retailer are as follows:

(12) $$\left\{\begin{array}{l} p_{1}^{*}=-\frac{2b_{2}Z_{1}+\left(\beta_{1}+\beta_{2}\gamma \right)Z_{2}}{{\varphi \beta }_{1}^{2}-4b_{1}b_{2}+{\gamma \beta }_{2}^{2}+\beta_{1}\beta_{2}+\beta_{1}\beta_{2}\varphi \gamma }\\ p_{2}^{*}=-\frac{2b_{1}Z_{2}+\left(\beta_{2}+\beta_{1}\varphi \right)Z_{1}}{{\varphi \beta }_{1}^{2}-4b_{1}b_{2}+{\gamma \beta }_{2}^{2}+\beta_{1}\beta_{2}+\beta_{1}\beta_{2}\varphi \gamma } \end{array}\right.$$

where

$Z_{1}=\theta a-\beta_{1}v+b_{1}c-\beta_{2}\left(c-w\right)-\beta_{2}\gamma \left(\frac{\eta v^{2}}{2}+w\right)+\frac{\theta \sqrt{\varepsilon I_{m}}}{\varepsilon }$
$Z_{2}=b_{2}v-a\left(\theta -1\right)-\varphi \left(\beta_{1}c-b_{2}\left(c-w\right)\right)+b_{2}\left(w+\frac{\eta v^{2}}{2}\right)-\frac{\left(\theta -1\right)\sqrt{\varepsilon I_{m}}}{\varepsilon }$.

Therefore, the optimal utility functions of the manufacturer and retailer are as follows:

(13) $$\left\{\begin{array}{l} \begin{array}{ll} U_{M}^{*}= & \left[\left(w-c\right)+\gamma \left(p_{2}^{*}-w-c_{r}\right)\right]\left[\left(1-\theta \right)a-b_{2}\left(p_{2}^{*}-v\right)+\beta_{2}p_{1}^{*}+\left(1-\theta \right)\frac{1}{\sqrt{\varepsilon }}\sqrt{I_{m}}\right]\\ & +\left(p_{1}^{*}-c\right)\left[\theta a-b_{1}p_{1}^{*}+\beta_{1}\left(p_{2}^{*}-v\right)+\theta \frac{1}{\sqrt{\varepsilon }}\sqrt{I_{m}}\right]-I_{m}\; \end{array}\\ \begin{array}{ll} U_{R}^{*}= & \left[\left(p_{2}^{*}-w-c_{r}\right)+\varphi \left(w-c\right)\right]\left[\left(1-\theta \right)a-b_{2}\left(p_{2}^{*}-v\right)+\beta_{2}p_{1}^{*}+\left(1-\theta \right)\frac{1}{\sqrt{\varepsilon }}\sqrt{I_{m}}\right]\\ & +\varphi \left(p_{1}^{*}-c\right)\left[\theta a-b_{1}p_{1}^{*}+\beta_{1}\left(p_{2}^{*}-v\right)+\theta \frac{1}{\sqrt{\varepsilon }}\sqrt{I_{m}}\right]-\varphi I_{m} \end{array} \end{array}\right..$$

Analyzing the optimal utility functions, three propositions can be obtained.

**Proposition** **1.** *The optimal pricings of the manufacturer and retailer increase with $I_{m}$.*

Proof is in Appendix A.

**Proposition** **2.** *The utility of the manufacturer is positively correlated with $I_{m}$ in the (0, $I_{m}^{\Delta }$), and is negatively correlated with $I_{m}$ in the ($I_{m}^{\Delta },I_{MAX}$).*

Proof is in Appendix A.

**Proposition** **3.** *In order to guarantee the profit of the retailer, the retailer will cooperate with the manufacturer when.*

$$\gamma \in (\frac{2b_{1}Z_{2}+\left(b_{2}+\varphi b_{1}\right)[\theta a-\beta_{2}\left(c-w\right)+\beta_{1}v+b_{1}c+\frac{\theta \sqrt{\varepsilon I_{m}}}{\varepsilon }]-\left(w+c_{r}\right)\left(\varphi \beta_{1}^{2}-4b_{1}b_{2}+\beta_{1}\beta_{2}\right)}{\left(w+c_{r}\right)\left(\beta_{2}^{2}+\varphi \beta_{1}\beta_{2}\right)+\left(b_{2}+\varphi b_{1}\right)\left(\beta_{2}c_{r}+\beta_{2}w\right)},1).$$

Proof is in Appendix A.

Due to the complexity of the external environment, the dual-channel value chain system is in a non-equilibrium state in most cases. The manufacturer and the retailer cannot know the full information each other and show bounded rational behavior. They make decisions of period t+1 based on marginal profit of period t. When the marginal profit in period t is positive, they will increase the price of period t+1 to gain more profit; when the marginal profit is negative, they will decrease the price of period t+1.

The marginal profits of the manufacturer and retailer are:

(14) $$\left\{\begin{array}{l} \begin{array}{ll} \frac{\partial U_{m}\left(t\right)}{\partial p_{1}\left(t\right)} & =\theta a-2b_{1}p_{1}\left(t\right)+\beta_{1}\left(p_{2}\left(t\right)-v_{r}\right)+\theta \frac{\sqrt{\varepsilon I_{m}}}{\varepsilon }+b_{1}c+\left(w-c\right)\beta_{2}\\ & +\gamma \left(p_{2}\left(t\right)-w-c_{r}\right)\beta_{2} \end{array}\\ \begin{array}{ll} \frac{\partial U_{r}\left(t\right)}{\partial p_{2}\left(t\right)} & =\left(1-\theta \right)a-2b_{2}p_{2}\left(t\right)+b_{2}v_{r}+\beta_{2}p_{1}\left(t\right)+\left(1-\theta \right)\frac{\sqrt{\varepsilon I_{m}}}{\varepsilon }+b_{2}\left(w+\frac{\eta v_{r}^{2}}{2}\right)\\ & +\varphi \left(p_{1}\left(t\right)-c\right)\beta_{1}-\varphi \left(w-c\right)b_{2} \end{array} \end{array}\right..$$

Rational expectation refers to the expectation of an economic phenomenon. If people are rational, they will make the most of the information they have to make actions without making systematic mistakes. However, the decision-maker’s ability to calculate and understand the environment is limited, so it is impossible for the decision-maker to know everything and show bounded rational behavior. The bounded rational expectation (BRE) is widely used to describe the dynamic decision process in the economic system (Li and Li, 2016; Zhang and Wang, 2017; Ma et al., 2017). Overall, the dynamic game model is:

(15) $$\left\{\begin{array}{l} p_{1}\left(t+1\right)=p_{1}\left(t\right)+\xi_{1}p_{1}\left(t\right)\frac{\partial U_{m}\left(p_{1}\left(t\right),p_{2}\left(t\right)\right)}{\partial p_{1}\left(t\right)}\\ p_{2}\left(t+1\right)=p_{2}\left(t\right)+\xi_{2}p_{2}\left(t\right)\frac{\partial U_{r}\left(p_{1}\left(t\right),p_{2}\left(t\right)\right)}{\partial p_{2}\left(t\right)} \end{array}\right..$$

Namely

(16) $$\left\{\begin{array}{l} \begin{array}{ll} p_{1}\left(t+1\right)= & p_{1}\left(t\right)+\xi_{1}p_{1}\left(t\right)\left[\theta a-2b_{1}p_{1}\left(t\right)+\beta_{1}\left(p_{2}\left(t\right)-v\right)+\theta \frac{\sqrt{\varepsilon I_{m}}}{\varepsilon }+b_{1}c\right]\\ & +\xi_{1}p_{1}\left(t\right)\left[+\left(w-c\right)\beta_{2}+\gamma \left(p_{2}\left(t\right)-w-c_{r}\right)\beta_{2}\right] \end{array}\\ \begin{array}{ll} p_{2}\left(t+1\right)= & p_{2}\left(t\right)+\xi_{2}p_{2}\left(t\right)\left[\left(1-\theta \right)a-2b_{2}p_{2}\left(t\right)+b_{2}v+\beta_{2}p_{1}\left(t\right)+\left(1-\theta \right)\frac{\sqrt{\varepsilon I_{m}}}{\varepsilon }\right]\\ & +\xi_{2}p_{2}\left(t\right)\left[b_{2}\left(w+\frac{\eta v^{2}}{2}\right)+\varphi \left(p_{1}\left(t\right)-c\right)\beta_{1}-\varphi \left(w-c\right)b_{2}\right] \end{array} \end{array}\right.$$

where $\xi_{1}\;\mathrm{and}\;\xi_{2}$ are the adjustment parameters of finite rationality; $p_{1}\;\mathrm{and}\;p_{2}$ are the decision variables in the dynamic model.

### 4.1. Equilibrium Points and Local Stability

The dynamic system (16) is nonlinear, what is different from the linear system is that the nonlinear system has multiple equilibriums. By setting $p_{i}\left(t+1\right)=p_{i}\left(t\right)$, the four equilibrium points of the dynamic system (16) can obtain:

$$E_{0}=\left(0,0\right),\;E_{1}=\left(\frac{Z_{1}}{2b_{1}},0\right),\;E_{2}=\left(0,\frac{Z_{2}}{2b_{2}}\right)\;E_{3}=\left(-\frac{2b_{2}Z_{1}+\left(\beta_{1}+\beta_{2}\gamma \right)Z_{2}}{\varphi \beta_{1}^{2}-4b_{1}b_{2}+\gamma \beta_{2}^{2}+\beta_{1}\beta_{2}+\beta_{1}\beta_{2}\varphi \gamma },-\frac{2b_{1}Z_{2}+\left(\beta_{2}+\beta_{1}\varphi \right)Z_{1}}{\varphi \beta_{1}^{2}-4b_{1}b_{2}+\gamma \beta_{2}^{2}+\beta_{1}\beta_{2}+\beta_{1}\beta_{2}\varphi \gamma }\right).$$

The Jacobian matrix of the dynamic system (16) is:

(17) $$J=\left[\begin{array}{ll} J_{11} & J_{12}\\ J_{21} & J_{22} \end{array}\right]$$

where

$J_{11}=1+\xi_{1}\left(Z_{1}-4b_{1}p_{1}+\beta_{1}p_{2}+\gamma \beta_{2}p_{2}\right)$,
$J_{12}=\left(\beta_{1}+\gamma \beta_{2}\right)\xi_{1}p_{1}$,
$J_{21}=\left(\beta_{2}+\varphi \beta_{1}\right)\xi_{2}p_{2}$,
$J_{22}=1+\xi_{2}\left(Z_{2}-4b_{2}p_{2}+\beta_{2}p_{1}+\varphi \beta_{1}p_{1}\right)$.

**Proposition** **4.** *The dynamic system (16) is stable at $E_{3}$ and is unstable at $E_{0},E_{1},E_{2}$*

Proof is in Appendix A.

The Jacobian matrix of $E_{3}$ is as follows:

(18) $$J\left(E_{3}\right)=\left[\begin{array}{ll} J_{11}^{*} & J_{12}^{*}\\ J_{21}^{*} & J_{22}^{*} \end{array}\right].$$

where

$J_{11}^{*}=1+Z_{1}\xi_{1}+\frac{8\xi_{1}b_{1}b_{2}Z_{1}+2\xi_{1}H_{1}b_{1}Z_{2}-\xi_{1}H_{1}H_{2}Z_{1}}{H_{3}}$,
$J_{12}^{*}=\frac{\xi_{1}H_{1}\left(2Z_{1}b_{2}+H_{1}Z_{2}\right)}{H_{3}}$,
$J_{21}^{*}=\frac{\xi_{2}H_{2}\left(2b_{1}Z_{2}+H_{2}Z_{1}\right)}{H_{3}}$,
$J_{22}^{*}=1+\xi_{2}Z_{2}+\frac{8\xi_{2}b_{1}b_{2}Z_{2}+2\xi_{2}H_{1}b_{2}Z_{1}-\xi_{2}H_{1}H_{2}Z_{2}}{H_{3}}$,
$H_{1}=\beta_{1}+\gamma \beta_{2}$,
$H_{2}=\beta_{2}+\varphi \beta_{1}$,
$H_{3}=\varphi \beta_{1}^{2}-4b_{1}b_{2}+\gamma \beta_{2}^{2}+\beta_{1}\beta_{2}+\varphi \gamma \beta_{1}\beta_{2}$.

The characteristic polynomial for the Jacobian matrix (20) takes the form:

(19) $$f\left(\lambda \right)=\lambda^{2}+K_{1}\lambda +K_{0}$$

where $\left\{\begin{array}{c} K_{1}=-\left(J_{11}^{*}+J_{22}^{*}\right)\\ K_{0}=J_{11}^{*}J_{22}^{*}-J_{12}^{*}J_{21}^{*} \end{array}\right.$.

The condition to maintain the stability of the dynamic system (16) can be described as:

(20) $$\left\{\begin{array}{l} \left(i\right):1+K_{1}+K_{0}>0\\ \left(ii\right):1-K_{1}+K_{0}>0\\ \left(iii\right):1-\left|K_{0}\right|>0 \end{array}\right..$$

When the adjustment parameters meet the above restrictions, the dynamic system (16) is in a stable state. The boundary value range of the adjustment parameters is as follows:

(21) $$\left\{\begin{array}{l} 0<\xi_{1}<-\frac{2H_{3}}{(H_{3}+8b_{1}b_{2}-H_{1}H_{2})Z_{1}+2H_{1}b_{1}Z_{2}}\\ 0<\xi_{2}<-\frac{2H_{3}}{\left(H_{3}+8b_{1}b_{2}-H_{1}H_{2}\right)Z_{2}+2H_{1}b_{2}Z_{1}} \end{array}\right..$$

### 4.2. Numerical Simulation

Nonlinear dynamics are related to many disciplines, such as mechanics, mathematics, physics, chemistry, and even some social sciences. They mainly involve three aspects: bifurcation, chaos, and soliton. In reality, the dynamic value chain system is a nonlinear system. Next, we will use the nonlinear dynamics theory to study the operation characteristics of a dynamic value chain system.

In this section, considering the complexity of dynamic system (16), the influence of parameters on the stability of dual-channel value chain system is studied by using the numerical simulation. According to the current situation of the green dual-channel value chain and other literature, the corresponding values of the parameters are as follows: $a=120,\;\theta =0.4,\;b_{1}=3,\;b_{2}=3.5,\;\beta_{1}=1,\;\beta_{2}=1,\;I_{m}=8,\;\varepsilon =1.2,\;v=2,\;\eta =0.5,\;c=6,\;w=10$. Thus, the Nash equilibrium point is $\left(p_{1}^{*},p_{2}^{*}\right)=\left(15.3178,18.8743\right)$.

#### 4.2.1. The Stability Region of a Dynamic System (16)

When parameter values of the power system are limited to a certain range, the system will behave as a stable state, which is a stable region; when parameter values of the power system exceed a certain range, the system will behave as a disordered state, which is an unstable region.

**Figure 2:**
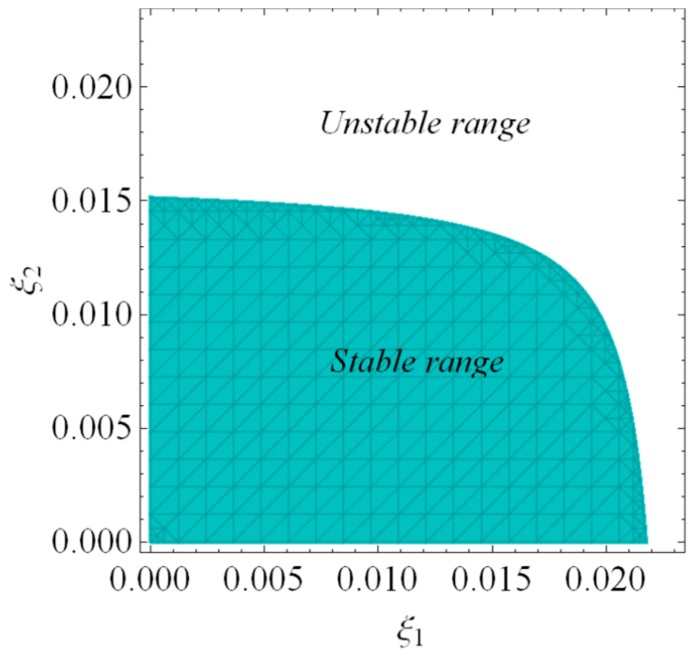
The stable region of Nash equilibrium point $E_{3}$.

According to inequality (20), Figure 2 depicts the stable region and unstable region of dynamic system (16). When $\xi_{1}$ and $\xi_{2}$ are employed in a stable range, the dynamic system (16) will tend to the equilibrium state after many iterations; when $\xi_{1}$ and $\xi_{2}$ are in an unstable range, the dynamic system (16) will enter into periodic motion or chaos after many iterations.

**Figure 3:**
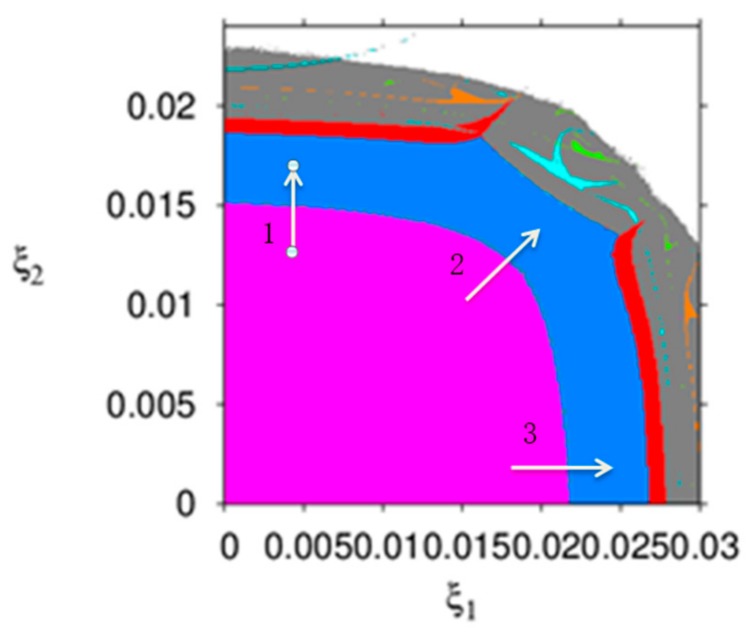
The evolution process of the dynamic system (16).

Figure 3 describes the parameter basin of dynamic system (16) with respect to $\xi_{1}$ and $\xi_{2}$, in which different colors represent different operation states of dynamic system (16), for example, the stable region (wine red), 2-period (blue), 4-period (red), 8-period (cyan), chaos (gray), and divergence (white). From Figure 3, the dynamic system (16) goes into chaos through period bifurcation (along arrows 1 and 3) or through Neimark–Sacker bifurcation (along arrows 2). If the price adjustment speeds are in the white region, the manufacture or the retailer will withdraw from the product market.

It can be seen from Figure 2 and Figure 3 that the smaller and appropriate price adjustment speed is conducive to the stable state of the market. With the price adjustment speed increasing, the market enters a chaotic state and unpredictable.

**Figure 4:**
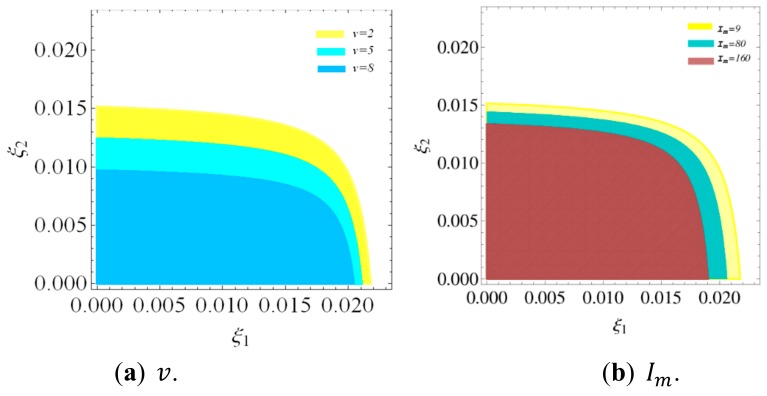

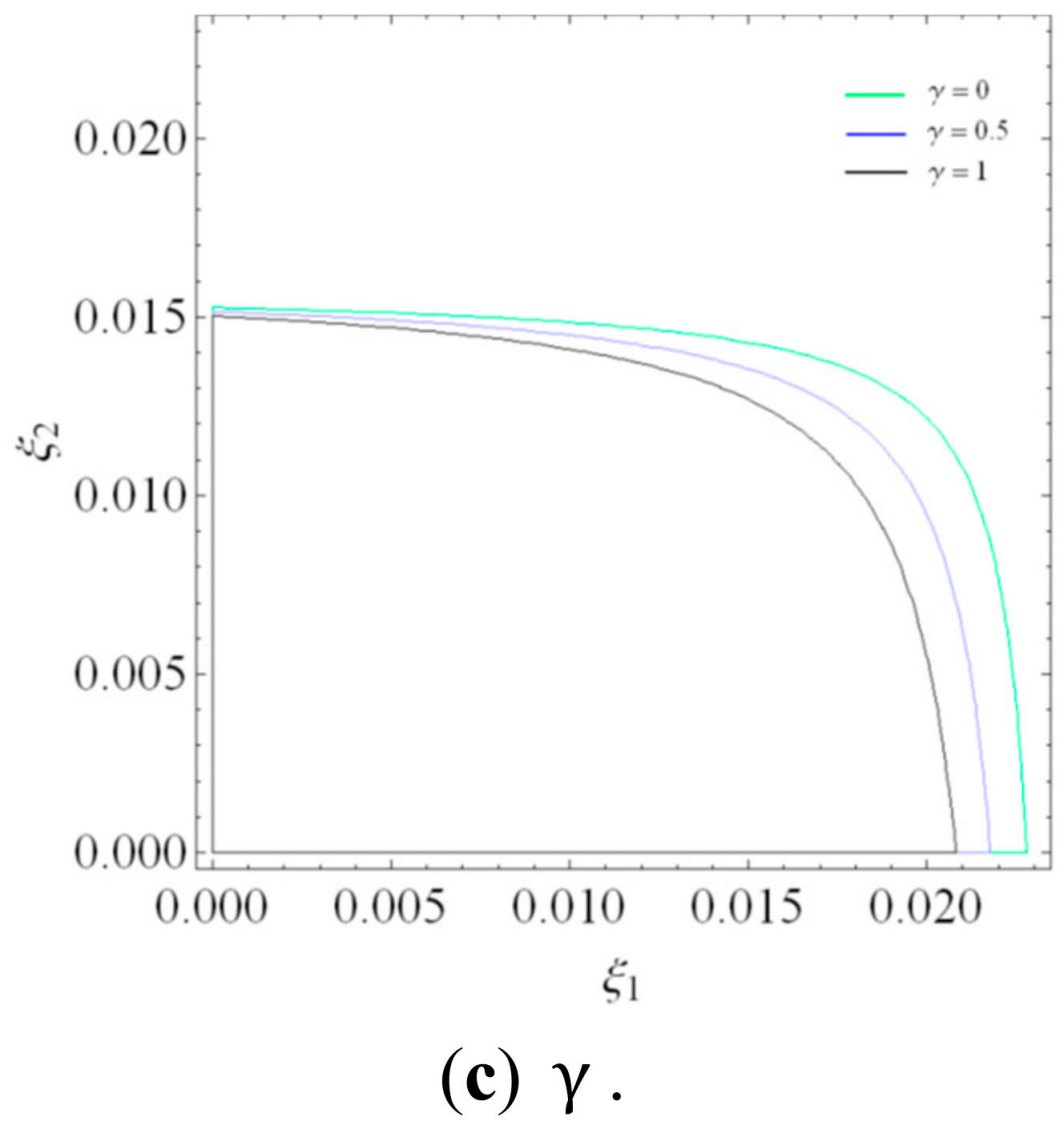
The stability regions of dynamic system (16) with v, $I_{m}$, and γ changing.

Next, we analyze the influence of v, $I_{m}$, and γ on the stability region of a dynamic system (16). Figure 4 shows the stability regions of a dynamic system (16) with v, $I_{m}$, and γ changing, respectively. According to Figure 4, the stability regions of dynamic system (16) decreases with v, $I_{m}$, and γ increasing.

Therefore, the decision making of the manufacturer and retailer are affected not only by their own behaviors, but also by the behaviors of competitors. So the manufacturer and retailer can work together to make reasonable investment strategies of price, green innovation input, and channel service to improve system stability and achieve optimal utility.

#### 4.2.2. The Influence of Price Adjustment Speed on the Dynamic System (16)

In addition to the equilibrium state, the stable steady state of the dynamic system also has periodic state, i.e., vibration, and a slightly more complex quasi periodic state. Parameter changing from large to small or from small to large sometimes causes the system changes from a stable state to periodic state and vice versa. Next, the bifurcation diagram and Lyapunov index are used to represent the evolution state of the dual-channel value system.

**Figure 5:**
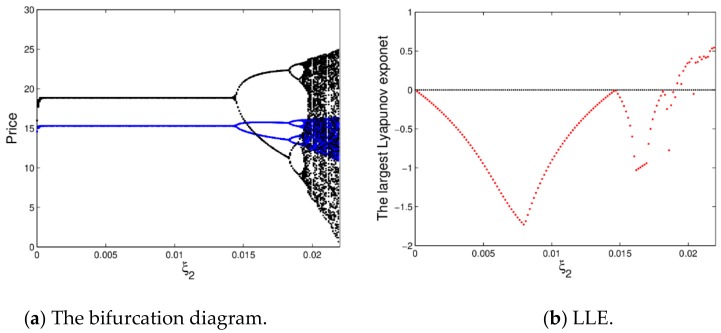
The bifurcation diagram and largest Lyapunov exponent (LLE) of the dynamic system (16).

Figure 5 shows the bifurcation diagram and the largest Lyapunov exponent (LLE) of dynamic system (16) with $\xi_{2}$ changing. From Figure 5a, the prices will be stable at the Nash equilibrium point when $\xi_{2}\in \left(0,\;0.014\right)$ with price fluctuations over several cycles, after that, the prices enter into chaotic state through flip bifurcation with $\xi_{2}$ increasing.

Figure 5b is accordance with the change process of Figure 5a, it shows the corresponding largest Lyapunov exponent (LLE) with $\xi_{2}$ varying from 0 to 0.022. When $\xi_{2}\leq 0$, the dynamic system (16) is in a stable state or period doubling bifurcation; when $\xi_{2}>0$, the system goes into a chaotic state.

Therefore, when the price adjustment speed is larger, it is not conducive to the stability of the market, while the smaller appropriate price adjustment is beneficial to the manufacturer and retailer’s profits to maintain the stability of the market.

When the system is in chaos, the chaotic attractor and the sensitivity to initial values are the most obvious features of chaotic system. An attractor is the steady state of value chain after long-term evolution. When the system has two very small initial values, after a long-term evolution of the system, the behavior of the system will be very different, showing the sensitivity to the initial value.

**Figure 6:**
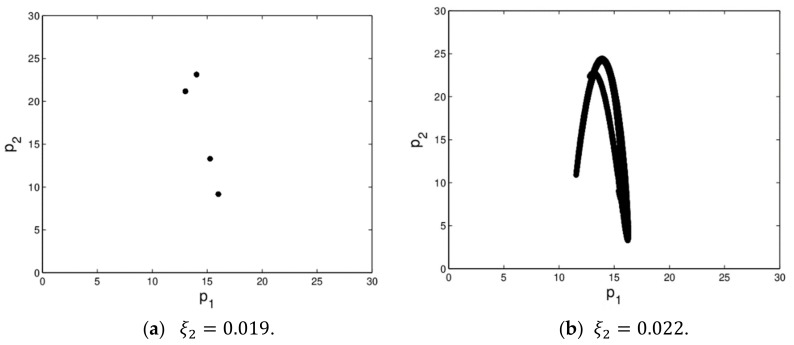
The attractors of dynamic system (16) when $\xi_{1}=0.01$.

The dynamic system (16) is in chaotic state when $\xi_{1}=0.01$ and $\xi_{2}=0.022$. Figure 6 shows the dynamic system (16) is in quadruple periodic bifurcation state and chaotic state. In chaotic state, the prices of the market changes irregularly, the operation process of dynamic system (16) becomes more complex which is not conducive to managers’ decision-making.

**Figure 7:**
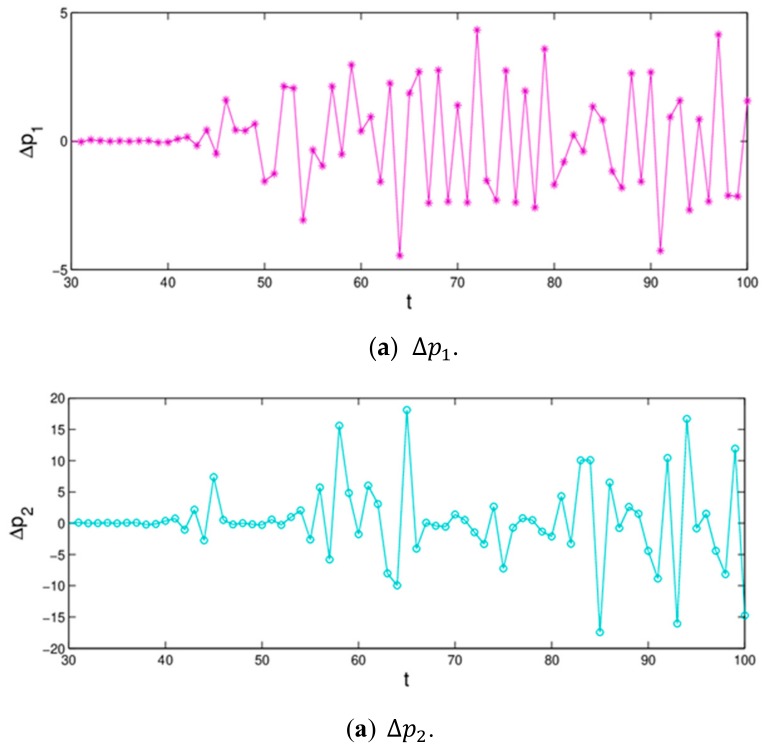
Sensitivity to initial value when $p_{2}$ changes 0.0001.

Figure 7 shows the differences of evolution process of dynamic system (16) when the initial value of $p_{2}$ only changes 0.0001. We can see that, the price values are no different in the first 43 iterations; however, after that, the differences of retail prices increase greatly. That is to say, the dynamic system (16) is very sensitive to the initial value in a chaotic state; a small change in initial value causes a huge deviation after multiple iterations, which give us reassurance that decision-makers should choose the initial values of their decision variables more prudently.

**Figure 8:**
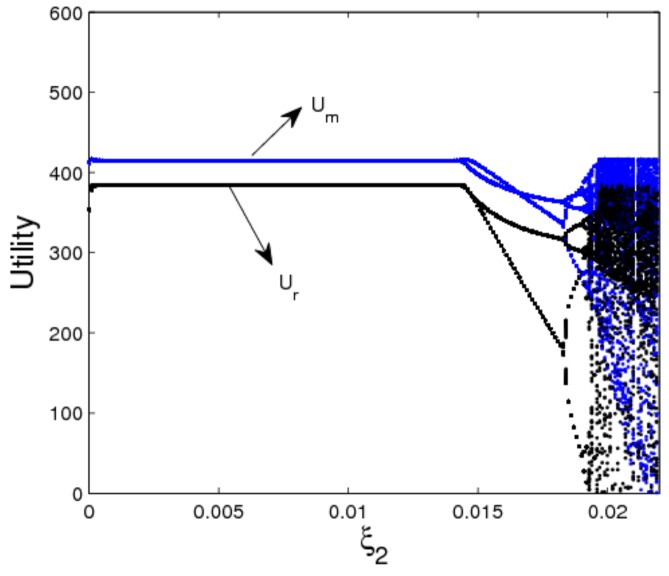
The utility evolution of a dynamic system (16) in terms of $\xi_{2}$ with $\xi_{1}=0.01$.

Figure 8 shows the utility evolution of the manufacturer and retailer when $\xi_{2}$ changes. In a stable state, the utility of the manufacturer is greater than that of the retailer as shown in Figure 8. When $0<\xi_{2}<0.014$, the dynamic system (16) is in a stable state, the manufacturer and retailer can get optimal utility; when $\xi_{2}\geq 0.014$, the dynamic system (16) is in an unstable state, the utilities of dynamic system (16) lose stability which is not conducive for participants to achieve business goals. The above analysis shows that the smaller appropriate price adjustment is beneficial to the manufacturer and retailer to make long-term plans and achieve long-term stable utility.

#### 4.2.3. The Influence of $I_{m}$ and v on the Utility of Dynamic Systems (16)

**Figure 9:**
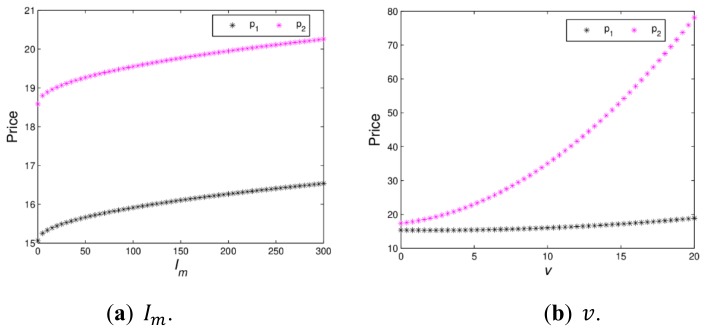
The price evolution of dymamic system (16) with $I_{m}$ and v increasing.

Figure 9 shows the changing trend of price with green innovation input and channel service increasing when a dynamic system (16) is in a stable state. When v=2, Figure 9a indicates that $p_{1}$ and $p_{2}$ increase with $I_{m}$, varying from 0 to 300. At the beginning, the retail prices of the manufacturer and retailer are growing rapidly, when $I_{m}>50$, the retail prices of the manufacturer and retailer grow slowly. Figure 9b shows that $p_{1}$ is greatly affected and $p_{2}$ is minimally affected with v increasing from 0 to 20. Therefore, appropriate green innovation inputs and channel service will help the manufacturer and retailer to obtain more profits. It is necessary for the manufacturer and the retailer to invest in green innovation input and channel service reasonably.

**Figure 10:**
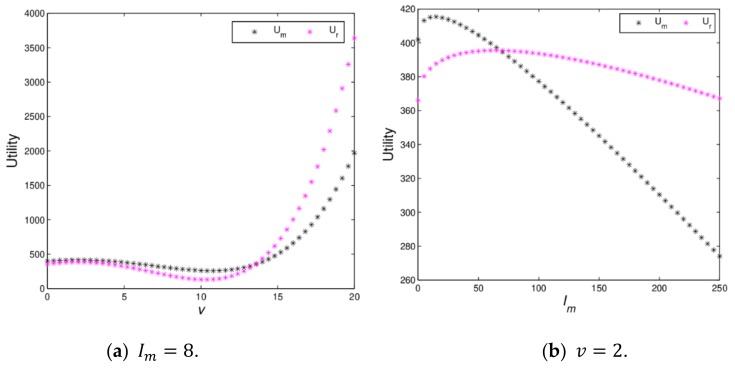
The utilities with change of v and $I_{m}$.

Figure 10 shows the utility changes of the manufacturer and retailer with $I_{m}$ and v varying when the dynamic system (16) is in stable state. Figure 10a indicates that the utilities of the manufacturer and retailer firstly decrease and then increase; when v>13.46, the utility of the retailer will be greater than that of manufacturer. Figure 10b shows that the utilities of the manufacturer and retailer firstly increase and then decrease with $I_{m},$ increasing from 0 to 250, which proves Proposition 2. When green innovation input exceeds a certain value, it will lead to a decline in utilities of the manufacturer and retailer, which is not conducive to the realization of the firm’s business objectives. The manufacturer should maintain appropriate green innovation inputs conducive to the development of enterprises.

**Figure 11:**
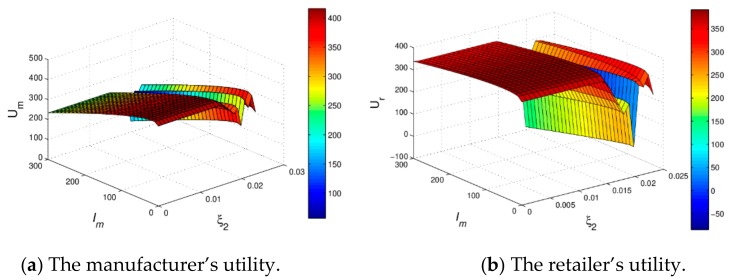
The 3D utility diagrams with $\xi_{2}$ and $I_{m}$ when $\xi_{1}=0.01$ and v=2.

Figure 11 shows the 3D utility evolution of dyanmic system (16) with $I_{m}$ and $\xi_{2}$ increasing, we can find that when $I_{m}$ changes from 0 to 300, the utilities of the manufacturer and retailer firstly increase and then decrease; when $\xi_{2}$ increase, the utilities of the manufacturer and the retailer will decrease and become unpredictable. For the decision makers, the reasonable green innovation input and the small price adjustment speed will help to obtain more profits, however they will not be able to achieve stable profits as green innovation input and the price adjustment speed increase, which is consistent with the static model.

## 5. Centralized Game Model

In this model, the objective of the manufacturer and the retailer is to maximize the total profit of dual-channel value chain. The total profit function is expressed as follows:

(22) $$\begin{array}{ll} \Pi^{c}= & \left(p_{2}-c-\frac{\eta v^{2}}{2}\right)\left[\left(1-\theta \right)a-b_{2}\left(p_{2}-v\right)+\beta_{2}p_{1}+\frac{1-\theta }{\sqrt{\varepsilon }}\sqrt{I_{m}}\right]\\ & +\left(p_{1}-c\right)\left[\theta a-b_{1}p_{1}+\beta_{1}\left(p_{2}-v\right)+\frac{\theta }{\sqrt{\varepsilon }}\sqrt{I_{m}}\right]-I_{m} \end{array}$$

Taking the first-order partial derivatives of $\prod^{c}$ with respect to $p_{1}\;$ and $p_{2}$, we can obtain the following equations:

(23) $$\left\{\begin{array}{l} \frac{\partial \prod^{c}}{\partial p_{1}}=-2b_{1}p_{1}+\left(\beta_{1}+\beta_{2}\right)p_{2}+a\theta -\beta_{1}v+b_{1}c-\frac{\eta \beta_{2}v^{2}}{2}-\beta_{2}c+\frac{\theta \sqrt{\varepsilon I_{m}}}{\varepsilon }\\ \frac{\partial \prod^{c}}{\partial p_{2}}=-2b_{2}p_{2}+\left(\beta_{1}+\beta_{2}\right)p_{1}-\beta_{1}c+b_{2}v-a\left(\theta -1\right)+\frac{b_{2}\eta v^{2}}{2}+b_{2}c-\frac{\left(\theta -1\right)\sqrt{\varepsilon I_{m}}}{\varepsilon } \end{array}\right.$$

The Hessian matrix of system (23) is as follows:

(24) $$H^{C}=\left[\begin{array}{cc} -2b_{1} & \beta_{1}+\beta_{2}\\ \beta_{1}+\beta_{2} & -2b_{2} \end{array}\right]$$

Since $b_{1}>0$, $b_{2}>0$, and $b_{i}>\beta_{i}$, i=1, 2

$$\left|H^{C}\right|=4b_{1}b_{2}-{\left(\beta_{1}+\beta_{2}\right)}^{2}>0$$

$\Pi^{c}$ is concave and has a unique maximum solution. Setting $\frac{\partial \prod^{c}}{\partial p_{1}}=0$, $\frac{\partial \prod^{c}}{\partial p_{2}}=0$, the optimal solutions are as follows:

(25) $$\left\{\begin{array}{l} p_{1}^{c}=-\frac{\beta_{1}s_{1}+\beta_{2}s_{1}+2b_{2}s_{0}}{\beta_{1}^{2}+2\beta_{1}\beta_{2}+\beta_{2}^{2}-4b_{1}b_{2}}\\ p_{2}^{c}=-\frac{\beta_{1}s_{0}+\beta_{2}s_{0}+2b_{1}s_{1}}{\beta_{1}^{2}+2\beta_{1}\beta_{2}+\beta_{2}^{2}-4b_{1}b_{2}} \end{array}\right.$$

where

$s_{0}=a\theta -\beta_{1}v+b_{1}c-\frac{\eta \beta_{2}v^{2}}{2}-\beta_{2}c+\frac{\theta \sqrt{\varepsilon I_{m}}}{\varepsilon }$
$s_{1}=-\beta_{1}c+b_{2}v-a\left(\theta -1\right)+\frac{b_{2}\eta v^{2}}{2}+b_{2}c-\frac{\left(\theta -1\right)\sqrt{\varepsilon I_{m}}}{\varepsilon }$

Substituting Equation (25) into Equation (22), we obtain the total optimal profit of dual-channel value chain.

(26) $$\Pi^{c}=\left(p_{2}^{c}-c-\frac{\eta v^{2}}{2}\right)\left[\left(1-\theta \right)a-b_{2}\left(p_{2}^{c}-v\right)+\beta_{2}p_{1}^{c}+\left(1-\theta \right)\frac{1}{\sqrt{\varepsilon }}\sqrt{I_{m}}\right]\;+\;\left(p_{1}^{c}-c\right)\left[\theta a-b_{1}p_{1}^{c}+\beta_{1}\left(p_{2}^{c}-v\right)+\theta \frac{1}{\sqrt{\varepsilon }}\sqrt{I_{m}}\right]-I_{m}$$

**Proposition** **5.** *The total profit of dual-channel value chain is positively correlated with $I_{m}$ within (0,$-\frac{L_{2}}{2L_{1}}$), and negatively correlated with $I_{m}$ within ($-\frac{L_{2}}{2L_{1}}$, $\frac{{\left(-L_{2}+\sqrt{L_{2}^{2}-4L_{1}L_{3}}\right)}^{2}}{4L_{1}^{2}}$).*

Proof is in Appendix A.

In this section, we develop a dynamic game model under the environment of centralized decision-making, in which the manufacturer and retailer all make decisions based on the limited rational expectation. Then, the long-term price forecasting system is as follows:

(27) $$\left\{\begin{array}{l} \begin{array}{ll} p_{1}\left(t+1\right)= & p_{1}\left(t\right)+\tau_{1}p_{1}\left(t\right)\left[-2b_{1}p_{1}\left(t\right)+\left(\beta_{1}+\beta_{2}\right)p_{2}\left(t\right)+a\theta -\beta_{1}v+b_{1}c-\frac{\eta \beta_{2}v^{2}}{2}\right]\\ & -\tau_{1}p_{1}\left(t\right)\beta_{2}c+\tau_{1}p_{1}\left(t\right)\frac{\theta \sqrt{\varepsilon I_{m}}}{\varepsilon } \end{array}\\ \begin{array}{ll} p_{2}\left(t+1\right)= & p_{2}\left(t\right)+\tau_{2}p_{2}\left(t\right)\left[-2b_{2}p_{2}\left(t\right)+\left(\beta_{1}+\beta_{2}\right)p_{1}\left(t\right)-\beta_{1}c-a\left(\theta -1\right)+\frac{b_{2}\eta v^{2}}{2}\right]\\ & +\tau_{2}p_{2}\left(t\right)(b_{2}c+b_{2}v)-\tau_{2}p_{2}\left(t\right)\frac{\left(\theta -1\right)\sqrt{\varepsilon I_{m}}}{\varepsilon } \end{array} \end{array}\right.$$

where $\tau_{i}\;(\tau_{i}>0,\;i=1,2)$ are the price adjustment speeds of the manufacturer and retailer.

### 5.1. Equilibrium Points and Local Stability

The four equilibrium points of dynamic system (27) are as follows:

$E_{0}=\left(0,0\right),\;E_{1}=\left(0,\frac{s_{1}}{2b_{2}}\right),\;E_{2}=\left(\frac{s_{0}}{2b_{1}},0\right),$
$E_{3}=\left(-\frac{\beta_{1}s_{1}+\beta_{2}s_{1}+2b_{2}s_{0}}{\beta_{1}^{2}+2\beta_{1}\beta_{2}+\beta_{2}^{2}-4b_{1}b_{2}},-\frac{\beta_{1}s_{0}+\beta_{2}s_{0}+2b_{1}s_{1}}{\beta_{1}^{2}+2\beta_{1}\beta_{2}+\beta_{2}^{2}-4b_{1}b_{2}}\right)$

**Proposition** **6.** *$E_{0},\;E_{1}\;and\;E_{2}$ are boundary equilibrium points, $E_{3}$ is the cooperation equilibrium point.*

Proof is in Appendix A.

The following analyzes the stable characteristics of the cooperative equilibrium point.

The Jacobian matrix of dynamic system (27) at $E_{3}$ is:

(28) $$J\left(E_{3}\right)=\left(\begin{array}{ll} J_{11}^{*} & J_{12}^{*}\\ J_{21}^{*} & J_{22}^{*} \end{array}\right)$$

where

$J_{11}^{*}=1+\frac{4b_{1}\tau_{1}\left(\beta_{1}s_{1}+\beta_{2}s_{1}+2b_{2}s_{0}\right)}{\beta_{1}^{2}+2\beta_{1}\beta_{2}+\beta_{2}^{2}-4b_{1}b_{2}}-\frac{\left(\beta_{1}+\beta_{2}\right)\tau_{1}\left(\beta_{1}s_{0}+\beta_{2}s_{0}+2b_{1}s_{1}\right)}{\beta_{1}^{2}+2\beta_{1}\beta_{2}+\beta_{2}^{2}-4b_{1}b_{2}}+\tau_{1}s_{0}$
$J_{12}^{*}=-\frac{\tau_{1}\left(\beta_{1}+\beta_{2}\right)\left(\beta_{1}s_{1}+\beta_{2}s_{1}+2b_{2}s_{0}\right)}{\beta_{1}^{2}+2\beta_{1}\beta_{2}+\beta_{2}^{2}-4b_{1}b_{2}}$
$J_{21}^{*}=-\frac{\tau_{2}\left(\beta_{1}+\beta_{2}\right)\left(\beta_{1}s_{0}+\beta_{2}s_{0}+2b_{1}s_{1}\right)}{\beta_{1}^{2}+2\beta_{1}\beta_{2}+\beta_{2}^{2}-4b_{1}b_{2}}$
$J_{22}^{*}=1+\frac{\left(\beta_{1}s_{0}+\beta_{2}s_{0}+2b_{1}s_{1}\right)4b_{1}\tau_{2}}{\beta_{1}^{2}+2\beta_{1}\beta_{2}+\beta_{2}^{2}-4b_{1}b_{2}}-\frac{\left(\beta_{1}+\beta_{2}\right)\left(\beta_{1}s_{1}+\beta_{2}s_{1}+2b_{2}s_{0}\right)\tau_{2}}{\beta_{1}^{2}+2\beta_{1}\beta_{2}+\beta_{2}^{2}-4b_{1}b_{2}}+\tau_{2}s_{1}$

The characteristic polynomial for the Jacobian matrix $J\left(E_{3}\right)$ is:

(29) $$f\left(\lambda \right)=\lambda^{2}+B\lambda +C$$

where $B=-\left(J_{11}^{*}+J_{22}^{*}\right)$, $C=J_{11}^{*}J_{22}^{*}-J_{12}^{*}J_{21}^{*}$.

According to July criterion, the necessary and sufficient condition for the locally stability of Nash equilibrium point $E_{3}$ is as follows:

(30) $$\left\{\begin{array}{l} \left(i\right):1+B+C>0\\ \left(ii\right):1-B+C>0\\ \left(iii\right):1-\left|C\right|>0 \end{array}\right.$$

In theory, we can get the stable range of dynamic system (27) by solving the inequality Equation (30), but inequality Equation (30) are too complicated to get a concrete expression. Next, the evolution characteristics of dynamic system (27) will be analyzed by numerical simulation.

### 5.2. Numerical Simulation

In this section, setting the same parameters values as the previous section, the cooperation equilibrium point is $E_{3}=\left(15.9,\;18.69\right)$.

#### 5.2.1. The Stability Region of Dynamic System (27)

**Figure 12:**
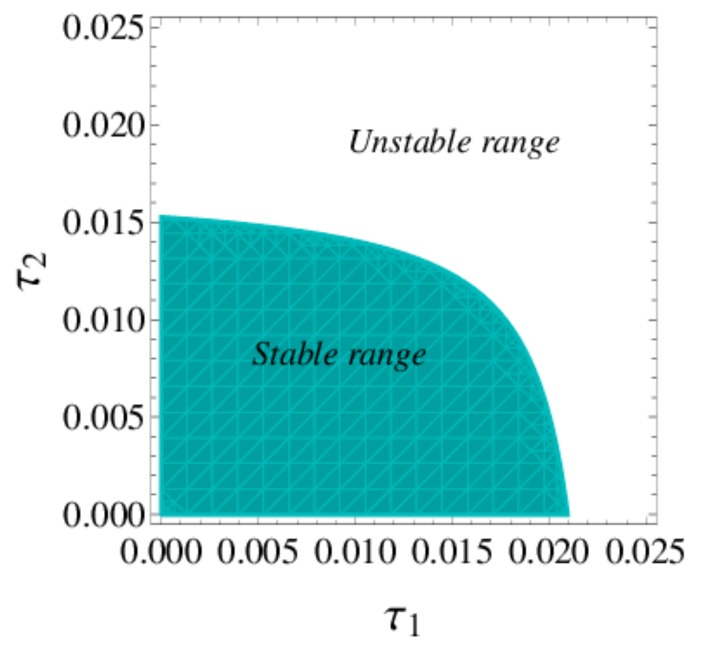
The stable region of dynamic system (27).

According to inequality Equation (30), Figure 12 depicts the stable region and unstable region of dynamic system (27) when v=2, $I_{m}=8$. When $\tau_{1}$ and $\tau_{2}$ are employed in the stable range, the dynamic system (27) will stabilize at cooperation equilibrium point after many games. When $\tau_{1}$ and $\tau_{2}$ go into unstable range, the dynamic system (27) will become unstable, which is unfavorable to the manufacturer and retailer. Compared with Figure 2, the stability region of the value chain in centralized decision-making is smaller than that in decentralized decision-making. With the decentralized decision made, a smaller price adjustment speed helps the market maintain a stable state.

**Figure 13:**
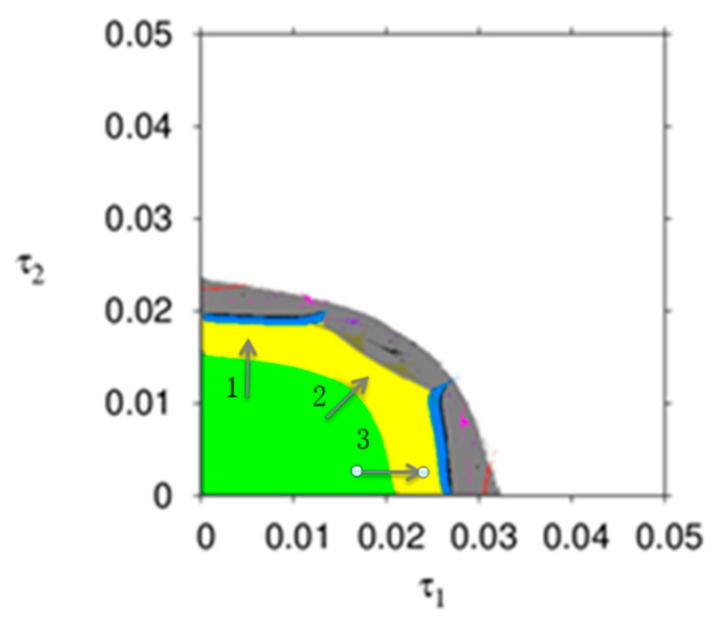
The parameter basins of dynamic system (27).

Figure 13 is the parameter basins of dynamic system (27) which can describe the evolution process of dynamic system (27) more intuitively, different colors represent different period, the stable region (green), 2-period (yellow), 4-period (blue), 8-period (black), chaos (gray) and divergence (white). From Figure 13, the dynamic system (16) goes into chaos through period bifurcation (along arrows 1 and arrows 3) or through Neimark-Sacker bifurcation (along arrows 2). If the price adjustment speeds are in the white region, the manufacture or the retailer will withdraw from the product market. It follows that the greater price adjustment speed is detrimental to the manufacturer and retailer.

From the managerial point of view, the participants’ price adjustment speed should be keep in stable range; otherwise, the dynamic system (27) will go into the chaotic state in which the prices is irregular, unpredictable, sensitive to initial values and is difficult for decision makers to make decisions.

**Figure 14:**
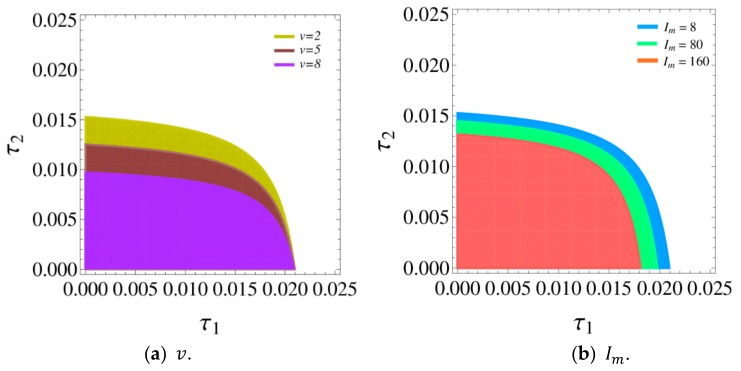
The stability regions of a dynamic system (27) with v and $I_{m}$ increasing.

Figure 14 is the stability regions of dynamic system (27) with v and $I_{m}$ having different values. In Figure 14a, with v increasing, the stable range of $\tau_{2}$ decreases, while that of $\tau_{1}$ remains unchanged. So the retailer increasing service level will narrow the stable range of his price adjustment and has no effect on that of the manufacturer. In Figure 14b, with $I_{m}$ increasing, the stability region of dynamic system (27) will decrease. Therefor the manufacturer increasing green innovation input will narrow the stable range of the manufacturer and retailer price adjustment.

The above analysis can draw clearly that the stability region of a dynamic system (27) is decreasing with green innovation input and service level increasing. Therefore, the manufacturer and retailer can work together to make reasonable green innovation input and service investment strategies to improve system’s stability and achieve optimal profitability.

#### 5.2.2. The Influence of Price Adjustment Speed on a Dynamic System (27)

**Figure 15:**
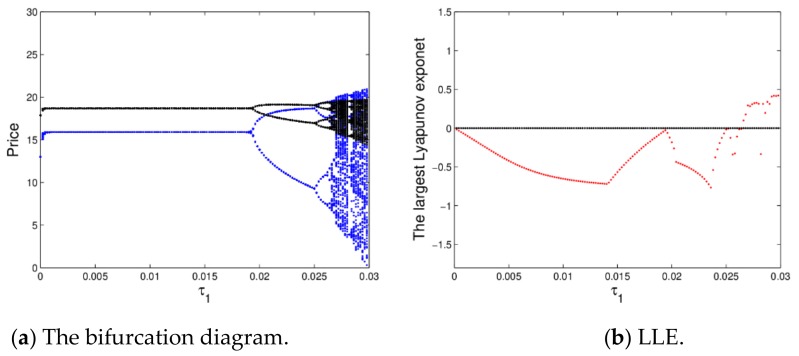
The bifurcation diagram and LLE of a dynamic system (27) when $\tau_{2}=0.01$.

Figure 15a shows the bifurcation diagram of a dynamic system (27) for $\tau_{2}=0.07$ and $\tau_{1}\in \left(0,0.03\right)$. When $\tau_{1}<0.0183$, the retail prices are in stable state; when $\tau_{1}>0.0183$, the dynamic system (27) occurs the flip bifurcation and then goes into chaos. The uncertain risk of the manufacturer and retailer will increase when the market is in chaotic state.

**Figure 16:**
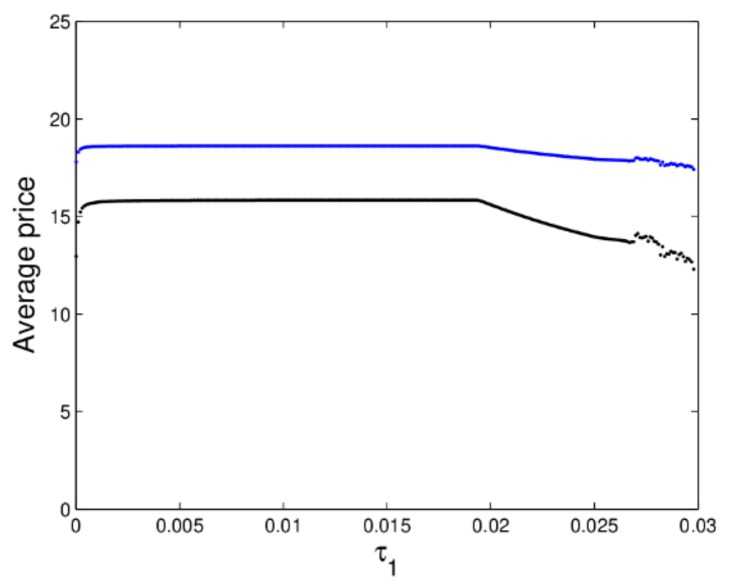
The average prices of dynamic system (27) with the change of $\tau_{1}$.

Figure 15b gives the corresponding largest Lyapunov exponent (LLE) of a dynamic system (27). When $\tau_{1}>0.0274$, the LLE of dynamic system (27) is almost positive, which shows the dynamic system (27) is in chaotic state. From Figure 16, we can know when the dynamic system (27) is in chaotic state, the average retail prices show a significant downward trend. The manufacturer and retailer can obtain a stable state under the appropriate price adjustment speed, as the price adjustment parameter increases, the market will lose stability.

**Figure 17:**
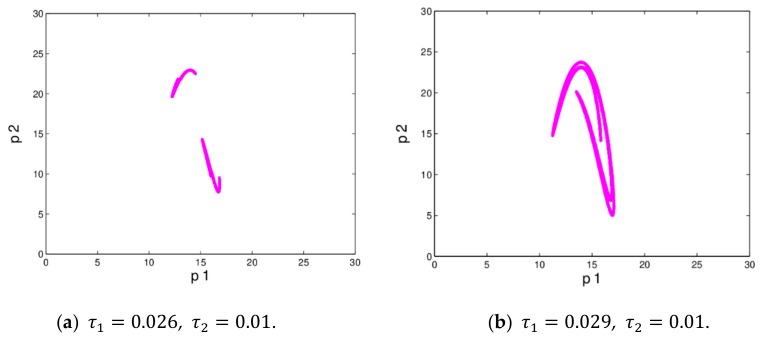
The chaotic attractors of dynamic system (27).

**Figure 18:**
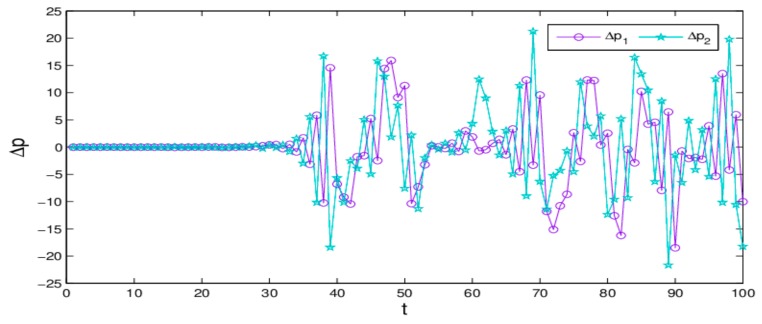
Sensitivity to initial value when $p_{1}$ changes 0.0001.

Figure 17 gives the chaotic attractors in different states. Figure 18 shows the differences of evolution process of dynamic system (27) when the initial value of $p_{1}$ are taken 13 and 13.001. We can see that the price values show no differences in the first 33 iterations; but after that, the differences of the retail prices increase greatly. That is to say, the dynamic system (27) is very sensitive to the initial value in a chaotic state; a small change in initial value will cause a huge deviation after multiple iterations, which give us reassurance that decision-makers should choose the initial values of their decision variables more prudently.

#### 5.2.3. The Influence of v and $I_{m}$ on a Dynamic System (27)

**Figure 19:**
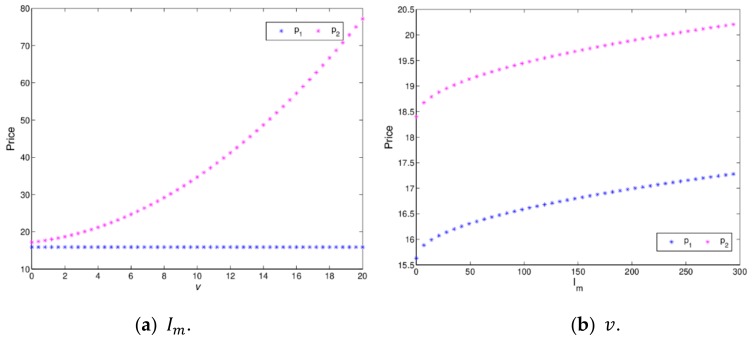
The price changes with $I_{m}$ and v changing.

Figure 19 shows the price changes with $I_{m}$ and v increasing. When $I_{m}=8$, the retail price of the retailer increase with v increasing, while the manufacture’s price is affected minimally, which is shown in Figure 19a. When v=2, Figure 19b indicates that the retail prices of the manufacturer and the retailer increase with $I_{m}$ varying. It means that retail prices increase with the service value and green innovation input increasing. So it is necessary for the manufacturer and retailer to invest in green innovation and channel service reasonably.

**Figure 20:**
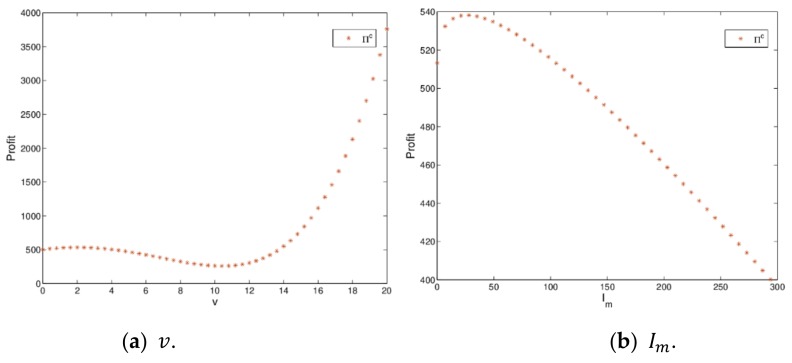
The total profit changes with v and $I_{m}$ changing.

Figure 20 shows the changing trend of total profit with v and $I_{m}$ varying. Figure 20a indicates that the total profit of a dynamic system (27) firstly decreases and then increases with v varying. Figure 20b shows that the total profit of a dynamic system (27) firstly increases and then decreases with $I_{m}$ increasing, it shows that when green innovation input has a certain value, decision makers can achieve the goal of maximizing profits (Proposition 5 is proved). Once green innovation input exceeds a certain value, it will cause a decline in the total profit and is not conducive to the realization of business objectives.

According to the Figure 19 and Figure 20, for the long-term development of the enterprise, it is necessary for the manufacturer and retailer to maintain appropriate green innovation input and channel service.

## 6. Conclusions

This paper constructs a dual-channel value chain composed of one manufacturer and one retailer where the manufacturer considers green innovation input for products and sells green products through the direct channel and the traditional channel. The retailer provides channel service input for customers and sells green products through the traditional channel. We consider two scenarios in which the manufacturer and the retailer make decentralized and centralized decisions, respectively. The conditions keeping the value chain system stable are found. The influences of parameters changing the pricing, profits, and stability of a dual-channel value chain are studied using a bifurcation diagram, LLE, and attraction of basin, etc. Some conclusions can be obtained: (1) the stability region of a dual-channel value chain in a decentralized decision is greater than that of a centralized decision and narrows with the increase of service value, green innovation input, and altruistic behavior. (2) With the price adjustment speed increasing, the dual-channel value chain enters into a chaotic state through flip bifurcation or N-S bifurcation. (3) In a stable state, the manufacturer and retailer can obtain the maximum utility choosing the appropriate value of green innovation input; in a chaotic state, the utilities of the manufacturer and retailer have been greatly affected and significantly reduced.

There are also some valuable managerial implications for decision makers in a dual-channel value chain. Firstly, decision makers should increase appropriate investment in green innovations and services to attract more consumers and achieve greater profits. Irrational green innovation input, however, will lead to a substantial loss of their own long-term profits. Secondly, when making price decisions, they need to grasp the market information in time and establish a reasonable price adjustment mechanism to avoid the chaos caused by too sudden price adjustments.

This paper has some limitations and can expand from the following aspects. Firstly, the impact of market demand randomness and the decision makers’ risk preference on the pricing and green innovation investment or service input of the value chain can be further studied. Secondly, the impact of the bullwhip effect on a dual-channel value chain remains an interesting topic to be studied.

## Figures and Tables

**Table 1:** Notations and their meaning used in this paper.

a	The Potential Market Size
θ	The preference coefficient for customer channel selection
w	The wholesale price
$p_{1}$	The retail price of direct channel
$p_{2}$	The retail price of traditional channel
c	The unit cost of the green product
$\xi_{i}$	The price adjustment speed under decentralized decision (i=1, 2)
Q	The demand caused by green innovation investment
$b_{i}$	The price elasticity coefficient (i=1, 2)
$\beta_{i}$	The cross-price elasticity coefficient (i = 1, 2)
$I_{m}$	The amount of green innovation investment
v	The service value of the traditional channel
$\tau_{i}$	The price adjustment speed under centralized decision (i=1, 2)
$c_{r}$	The service cost of the retailer
ε	The green innovation input parameter
γ	The altruistic preference coefficient of the manufacturer
φ	The altruistic preference coefficient of the retailer

## Appendix A

**Proof** **of** **Proposition** **1.** The optimal pricings of the manufacturer and retailer increase with $I_{m}$. Making

$\frac{\partial p_{1}^{*}}{\partial I_{m}}=-\frac{2b_{2}\theta \sqrt{\varepsilon I_{m}}+\left(\beta_{1}+\beta_{2}\gamma \right)\left(1-\theta \right)\sqrt{\varepsilon I_{m}}}{2\varepsilon I_{m}\left(\varphi \beta_{1}^{2}-4b_{1}b_{2}+\gamma \beta_{2}^{2}+\beta_{1}\beta_{2}+\beta_{1}\beta_{2}\varphi \gamma \right)}$,
$\frac{\partial p_{2}^{*}}{\partial I_{m}}=-\frac{2b_{1}\left(1-\theta \right)\sqrt{\varepsilon I_{m}}+\left(b_{2}+b_{1}\varphi \right)\theta \sqrt{\varepsilon I_{m}}}{2\varepsilon I_{m}\left(\varphi \beta_{1}^{2}-4b_{1}b_{2}+\gamma \beta_{2}^{2}+\beta_{1}\beta_{2}+\beta_{1}\beta_{2}\varphi \gamma \right)}$

According to the above restrictions on parameter values, we see $\varphi \beta_{1}^{2}-4b_{1}b_{2}+\gamma \beta_{2}^{2}+\beta_{1}\beta_{2}+\beta_{1}\beta_{2}\varphi \gamma <0$, so $\frac{\partial p_{1}^{*}}{\partial I_{m}}>0\;$ and $\frac{\partial p_{2}^{*}}{\partial I_{m}}>0$. Hence, the optimal prices increase as $I_{m}\;$ increases, the proof is complete. □

**Proof** **of** **Proposition** **2.** The utility of the manufacturer is positively correlated with $I_{m}$ in the (0, $I_{m}^{\Delta }$), and is negatively correlated with $I_{m}$ in the ($I_{m}^{\Delta },I_{MAX}$). In Equation (13), $U_{M}^{*}$ is a quadratic function with respect to $\sqrt{I_{m}}$. Letting $\frac{\partial U_{M}^{*}}{\partial \sqrt{I_{m}}}=0$, we can obtain

$$\begin{array}{ll} I_{m}^{\Delta } & =(\frac{\left(c+T_{1}\right)\left(b_{1}T_{0}-K_{3}\theta F+\beta_{1}T_{3}\right)-\left(c-w\right)\left(b_{2}T_{3}-K_{3}\left(\theta -1\right)+\beta_{2}T_{0}\right)}{2T_{0}(\frac{b_{1}T_{0}}{K_{3}}-F\theta +\frac{\beta_{1}T_{3}}{K_{3}})+2\gamma T_{3}(\frac{b_{2}T_{3}}{K_{3}}-F\left(\theta -1\right)+\frac{\beta_{2}T_{0}}{K_{3}})}\\ & {+\frac{-\gamma \left(\frac{\eta v^{2}}{2}+w+T_{2}\right)\left(b_{2}T_{6}-K_{3}F\left(\theta -1\right)+K_{3}T_{2}\right)+T_{3}\left(a\theta -\beta_{1}\left(v+T_{2}\right)+b_{1}T_{1}\right)}{2T_{0}(\frac{b_{1}T_{0}}{K_{3}}-F\theta +\frac{\beta_{1}T_{3}}{K_{3}})+2\gamma T_{3}(\frac{b_{2}T_{3}}{K_{3}}-F\left(\theta -1\right)+\frac{\beta_{2}T_{0}}{K_{3}})})}^{2} \end{array}$$

where: $F=\frac{1}{\sqrt{\varepsilon }}$

$K_{1}=a\theta -\beta_{2}\left(c-w\right)-\beta_{1}v+b_{1}c-\beta_{2}\gamma \left(\frac{\eta v^{2}}{2}+w\right)$
$K_{2}=b_{2}\left(\frac{\eta v^{2}}{2}+w\right)+b_{2}v-a\left(\theta -1\right)-\varphi \left(\beta_{1}c-b_{2}c+b_{2}w\right)$
$K_{3}=\varphi \beta_{1}^{2}-4b_{1}b_{2}+\beta_{2}^{2}\gamma +\beta_{1}\beta_{2}+\beta_{1}\beta_{2}\varphi \gamma$
$T_{0}=\beta_{1}F\left(\theta -1\right)-2Fb_{2}\theta +\beta_{2}F\gamma \left(\theta -1\right)$
$T_{1}=\frac{2K_{1}b_{2}+\beta_{1}K_{2}+\beta_{2}K_{2}r}{K_{3}}$
$T_{2}=\frac{2K_{2}b_{1}+\beta_{2}K_{1}+\varphi \beta_{1}K_{1}}{K_{3}}$
$T_{16}=\beta_{2}F\theta -2Fb_{1}\left(\theta -1\right)+\beta_{1}F\theta \varphi$

Letting $U_{M}^{*}\left(\sqrt{I_{m}}\right)=0$, and obtain

$$I_{MAX}=\frac{{\left(P_{7}-P_{4}+P_{2}C-P_{2}w+\sqrt{\begin{array}{c} 4P_{6}-4P_{3}+4P_{1}c-4P_{1}w+P_{4}^{2}+P_{7}^{2}+P_{2}^{2}c^{2}+P_{2}^{2}w^{2}-4P_{3}P_{5}\\ +4P_{3}P_{8}-2P_{4}P_{7}+4P_{5}P_{6}-4P_{6}P_{8}+4P_{1}P_{5}c-2P_{2}P_{4}c-4P_{1}P_{8}c\\ +2P_{2}P_{7}-4P_{1}P_{5}w+2P_{2}P_{4}w+4P_{1}P_{8}w-2P_{2}P_{7}w-2P_{2}^{2}cw \end{array}}\right)}^{2}}{4{\left(P_{5}-P_{8}+1\right)}^{2}}>0$$

$$I_{MIN}=-\frac{{\left(P_{7}-P_{4}+P_{2}C-P_{2}w-\sqrt{\begin{array}{c} 4P_{6}-4P_{3}+4P_{1}c-4P_{1}w+P_{4}^{2}+P_{7}^{2}+P_{2}^{2}c^{2}+P_{2}^{2}w^{2}-4P_{3}P_{5}\\ +4P_{3}P_{8}-2P_{4}P_{7}+4P_{5}P_{6}-4P_{6}P_{8}+4P_{1}P_{5}c-2P_{2}P_{4}c-4P_{1}P_{8}c\\ +2P_{2}P_{7}-4P_{1}P_{5}w+2P_{2}P_{4}w+4P_{1}P_{8}w-2P_{2}P_{7}w-2P_{2}^{2}cw \end{array}}\right)}^{2}}{4{\left(P_{5}-P_{8}+1\right)}^{2}}<0$$

where

$R_{1}=2b_{2}K_{1}+\beta_{1}K_{2}+\beta_{2}rK_{2}$
$R_{2}=2b_{2}\theta F-\beta_{1}\left(\theta -1\right)F-\beta_{2}\left(\theta -1\right)F$
$R_{3}=2b_{1}K_{2}+\beta_{2}K_{1}+\varphi \beta_{1}K_{1}$
$R_{4}=-2b_{1}\left(\theta -1\right)F+\beta_{2}\theta F+\varphi \beta_{1}\theta F$
$P_{1}=a\left(\theta -1\right)-b_{2}\gamma -\frac{b_{2}R_{3}}{K_{3}}+\frac{\beta_{2}R_{1}}{K_{3}}$
$P_{2}=-\frac{b_{2}R_{4}}{K_{3}}+\frac{\beta_{2}R_{2}}{K_{3}}-F\left(\theta -1\right)$
$P_{3}=a\theta \left(c+\frac{R_{1}}{K_{3}}\right)+\beta_{1}\gamma \left(c+\frac{R_{1}}{K_{3}}\right)-\frac{\beta_{1}R_{3}}{K_{3}}\left(c+\frac{R_{1}}{K_{3}}\right)+\frac{b_{1}R_{1}\beta_{1}}{K_{3}}$
$P_{4}=-\frac{\beta_{1}R_{4}}{K_{3}}\left(c+\frac{R_{1}}{K_{3}}\right)+\frac{b_{1}R_{2}}{K_{3}}\left(c+\frac{R_{1}}{K_{3}}\right)+F\theta \left(c+\frac{R_{1}}{K_{3}}\right)+\frac{a\theta R_{2}}{K_{3}}-\frac{\beta_{1}vR_{2}}{K_{3}}-\frac{R_{2}\beta_{1}R_{3}}{K_{3}^{2}}+\frac{R_{2}b_{1}R_{1}}{K_{3}^{2}}+\frac{R_{2}^{2}b_{1}}{K_{3}^{2}}$
$P_{5}=-\frac{\beta_{1}R_{2}R_{4}}{K_{3}^{2}}+\frac{R_{2}F\theta }{K_{3}}$
$P_{6}=\gamma \left(\frac{\eta v^{2}}{2}+w+\frac{R_{3}}{K_{3}}\right)\left(a\left(\theta -1\right)-b_{2}\gamma -\frac{b_{2}R_{3}}{K_{3}}+\frac{\beta_{2}R_{1}}{K_{3}}\right)$
$P_{7}=\gamma \left(\frac{\eta v^{2}}{2}+w+\frac{R_{3}}{K_{3}}\right)\left(-\frac{b_{2}R_{4}}{K_{3}}+\frac{\beta_{2}R_{2}}{K_{3}}+F\left(\theta -1\right)\right)+\frac{\gamma R_{4}}{K_{3}}\left(a\left(\theta -1\right)-b_{2}\gamma -\frac{b_{2}R_{3}}{K_{3}}+\frac{\beta_{2}R_{1}}{K_{3}}\right)$
$P_{8}=\frac{\gamma R_{4}}{K_{3}}\left(-\frac{b_{2}R_{4}}{K_{3}}+\frac{\beta_{2}R_{2}}{K_{3}}+F\left(\theta -1\right)\right)$

As $I_{m}>0$, we know $U_{M}^{*}$ is an incremental function in the range of $\left(I_{m}^{\Delta },I_{MAX}\right)$, and is a subtractive function in the range of $\left(I_{m}^{\Delta },I_{MAX}\right)$. Proposition 2 is proved. □

**Proof** **of** **Proposition** **3.** In order to guarantee the profit of the retailer, the retailer will cooperate with the manufacturer when

$$\gamma \in (\frac{2b_{1}Z_{2}+\left(b_{2}+\varphi b_{1}\right)[\theta a-\beta_{2}\left(c-w\right)+\beta_{1}v+b_{1}c+\frac{\theta \sqrt{\varepsilon I_{m}}}{\varepsilon }]-\left(w+c_{r}\right)\left(\varphi \beta_{1}^{2}-4b_{1}b_{2}+\beta_{1}\beta_{2}\right)}{\left(w+c_{r}\right)\left(\beta_{2}^{2}+\varphi \beta_{1}\beta_{2}\right)+\left(b_{2}+\varphi b_{1}\right)\left(\beta_{2}c_{r}+\beta_{2}w\right)},1).$$

The utility of the retailer will be positive when the sum of the manufacturer’s wholesale price and the retailer’s service cost is less than the retailer’s retail price. So, we will obtain the following inequality

$$w+c_{r}<p_{2}^{*}=-\frac{2b_{1}Z_{2}+\left(b_{2}+b_{1}\varphi \right)Z_{1}}{\varphi \beta_{1}^{2}-4b_{1}b_{2}+\gamma \beta_{2}^{2}+\beta_{1}\beta_{2}+\beta_{1}\beta_{2}\varphi \gamma }$$

By solving the above inequality, we know that:
$0<\frac{2b_{1}Z_{2}+\left(b_{2}+\varphi b_{1}\right)\left(\theta a-\beta_{2}\left(c-w\right)+\beta_{1}v+b_{1}c+\frac{\theta \sqrt{\varepsilon I_{m}}}{\varepsilon }\right)-\left(w+c_{r}\right)\left(\varphi \beta_{1}^{2}-4b_{1}b_{2}+\beta_{1}\beta_{2}\right)}{\left(w+c_{r}\right)\left(\beta_{2}^{2}+\varphi \beta_{1}\beta_{2}\right)+\left(b_{2}+\varphi b_{1}\right)\left(\beta_{2}c_{r}+\beta_{2}w\right)}<\gamma$
Since 0<γ<1, then, $\frac{2b_{1}Z_{2}+\left(b_{2}+\varphi b_{1}\right)\left(\theta a-\beta_{2}\left(c-w\right)+\beta_{1}v+b_{1}c+\frac{\theta \sqrt{\varepsilon I_{m}}}{\varepsilon }\right)-\left(w+c_{r}\right)\left(\varphi \beta_{1}^{2}-4b_{1}b_{2}+\beta_{1}\beta_{2}\right)}{\left(w+c_{r}\right)\left(\beta_{2}^{2}+\varphi \beta_{1}\beta_{2}\right)+\left(b_{2}+\varphi b_{1}\right)\left(\beta_{2}c_{r}+\beta_{2}w\right)}<\gamma <1$. Proposition 3 is proved. □

**Proof** **of** **Proposition** **4.** The dynamic system (16) is stable at $E_{3}$ and is unstable at $E_{0},E_{1},E_{2}$. At $E_{1}=\left(\frac{Z_{1}}{2b_{1}},0\right)$, the Jacobian matrix of dynamic system (16) is:

$$J\left(E_{1}\right)=\left(\begin{array}{cc} 1-\xi_{1}Z_{1} & \xi_{1}\left(\beta_{1}+\gamma \beta_{2}\right)\frac{Z_{1}}{2b_{1}}\\ 0 & 1+\xi_{2}\left[Z_{2}+\left(\beta_{2}+\varphi \beta_{1}\right)\frac{Z_{1}}{2b_{1}}\right] \end{array}\right)$$

The characteristic values of a dynamic system (16) at $E_{1}$ are:

$$\lambda_{1}=1-\xi_{1}Z_{1}\;\lambda_{2}=1+\xi_{2}Z_{2}+\xi_{2}\left(\beta_{2}+\varphi \beta_{1}\right)\frac{Z_{1}}{2b_{1}}.$$

As $Z_{2}>$, so $\lambda_{2}>1$, the equilibrium point $E_{1}$ is untable. Similarly, $E_{0}$ and $E_{2}$ are the unstable equilibrium solutions. Proposition 4 is proved. □

**Proof** **of** **Proposition** **5.** The total profit of dual-channel value chain is positively correlated with $I_{m}$ within (0,$-\frac{L_{2}}{2L_{1}}$), and negatively correlated with $I_{m}$ within ($-\frac{L_{2}}{2L_{1}}$, $\frac{{\left(-L_{2}+\sqrt{L_{2}^{2}-4L_{1}L_{3}}\right)}^{2}}{4L_{1}^{2}}$). Letting $\frac{\partial \Pi^{c}}{\partial \sqrt{I_{m}}}=0$, we obtain $I_{m}=-\frac{L_{2}}{2L_{1}}$. Setting $\Pi^{c}\left(I_{m}\right)=0$, we obtain $I_{m}^{min}=-\frac{{\left(L_{2}+\sqrt{L_{2}^{2}-4L_{1}L_{3}}\right)}^{2}}{4L_{1}^{2}}$ and $I_{m}^{max}=\frac{{\left(-L_{2}+\sqrt{L_{2}^{2}-4L_{1}L_{3}}\right)}^{2}}{4L_{1}^{2}}$. When $I_{m}\epsilon (I_{m}^{min},-\frac{L_{2}}{2L_{1}})$, the total profit of the value chain is positively, when $I_{m}\in (-\frac{L_{2}}{2L_{1}},I_{m}^{max})$, the total profit of the value chain is negative. Since $I_{m}>0$, the total profit of the value chain is positively correlated with $I_{m}$, in the interval (0, $-\frac{L_{2}}{2L_{1}}$), and it is negatively correlated with $I_{m}$ in the interval ($-\frac{L_{2}}{2L_{1}}$, $\frac{{\left(-L_{2}+\sqrt{L_{2}^{2}-4L_{1}L_{3}}\right)}^{2}}{4L_{1}^{2}}$). Where

$L_{1}=\theta Q_{2}\frac{\sqrt{\varepsilon }}{\varepsilon }-\left(e-1\right)Q_{4}\frac{\sqrt{\varepsilon }}{\varepsilon }-b_{1}Q_{2}^{2}-b_{2}Q_{4}^{2}+(\beta_{1}+\beta_{2})Q_{2}Q_{4}-1$
$L_{2}=Y_{1}Q_{2}+Q_{1}\theta \frac{\sqrt{\varepsilon }}{\varepsilon }+Y_{2}Q_{4}-Q_{3}\left(\theta -1\right)\frac{\sqrt{\varepsilon }}{\varepsilon }-2b_{1}Q_{1}Q_{2}-2b_{2}Q_{3}Q_{4}+\left(\beta_{1}+\beta_{2}\right)Q_{1}Q_{4}+(\beta_{1}+\beta_{2})Q_{2}Q_{3}+Y_{4}$
$L_{3}=Y_{1}Q_{1}+Y_{2}Q_{3}-b_{1}Q_{1}^{2}-b_{2}Q_{3}^{2}+(\beta_{1}+\beta_{2})Q_{1}Q_{3}+Y_{3}$
$Q_{1}=\frac{\left(4\beta_{1}+2\beta_{2}r-z_{1}^{2}-8b_{1}b_{2}\right)}{4b_{2}}$
$Q_{2}=\frac{\left(2\beta_{1}+\beta_{2}\right)z_{2}-z_{1}b_{2}-z_{1}z_{2}+4\beta_{2}b_{2}-2\beta_{2}b_{2}}{4b_{2}}$
$Q_{3}=\frac{2b_{2}\beta_{2}-2b_{2}\beta_{2}-b_{2}z_{1}+\left(2\beta_{1}+\beta_{2}\right)z_{2}-z_{1}z_{2}}{4b_{2}}$
$Q_{4}=\frac{z_{2}b_{2}-2z_{2}b_{2}-2b_{2}z_{2}+b_{2}z_{2}-z_{2}^{2}}{4b_{2}}$
$z_{1}=\frac{1}{2}\beta_{1}+\beta_{2}$; $z_{2}=\frac{1}{2}b_{2}$
$Y_{1}=\theta a-\beta_{1}v+b_{1}c-\beta_{2}\left(c+\frac{nv^{2}}{2}\right)$
$Y_{2}=\left(1-\theta \right)a-\beta_{1}c+b_{2}v+b_{2}\left(c+\frac{nv^{2}}{2}\right)$
$Y_{3}=-\theta ac+\beta_{1}cv-\left(b_{2}v+a-\theta a\right)\left(c+\frac{\eta v^{2}}{2}\right)$
$Y_{4}=-\theta c\frac{\sqrt{\varepsilon }}{\varepsilon }-\left(c+\frac{\eta v^{2}}{2}\right)\left(1-\theta \right)\frac{\sqrt{\varepsilon }}{\varepsilon }$.

The Proposition 5 is proved. □

**Proof** **of** **Proposition** **6.** $E_{0},\;E_{1}\;\mathrm{and}\;E_{2}$ are boundary equilibrium points, $E_{3}\;$ is the cooperation equilibrium point. The Jacobian matrix of dynamic system (27) at $E_{1}$ is as follows:

(31) $$J\left(E_{1}\right)=\left|\begin{array}{cc} 1+\tau_{1}(\left(\beta_{1}+\beta_{2}\right)\frac{s_{1}}{2b_{2}}+s_{0}) & 0\\ \tau_{2}\left(\beta_{1}+\beta_{2}\right)\frac{s_{1}}{2b_{2}} & 1+\tau_{2}\left(-\frac{4b_{1}s_{1}}{2b_{2}}+s_{1}\right) \end{array}\right|.$$

The eigenvalues at $E_{1}$ are as follows:

(32) $$\left\{\begin{array}{l} \lambda_{1}=1+\frac{\tau_{1}s_{1}\left(\beta_{1}+\beta_{2}\right)+2\tau_{1}b_{2}s_{0}}{2b_{2}}\\ \lambda_{2}=1-\frac{2\tau_{2}b_{1}s_{1}+\tau_{2}b_{2}s_{1}}{b_{2}} \end{array}\right..$$

Since $s_{0}>0,\;s_{1}>0,\;\beta_{i}>0,b_{i}>0,i=1,2$, we get $\lambda_{1}>1$, $\lambda_{2}<1$. Hence, the equilibrium point $E_{1}$ is unstable. The proofs of $E_{0}\;\mathrm{and}\;E_{2}$ are similar to that of $E_{1}$. Proposition 6 is proved. □
